# *Helicobacter pylori bab* Paralog Distribution and Association with *cagA*, *vacA*, and *homA/B* Genotypes in American and South Korean Clinical Isolates

**DOI:** 10.1371/journal.pone.0137078

**Published:** 2015-08-28

**Authors:** Aeryun Kim, Stephanie L. Servetas, Jieun Kang, Jinmoon Kim, Sungil Jang, Ho Jin Cha, Wan Jin Lee, June Kim, Judith Romero-Gallo, Richard M. Peek, D. Scott Merrell, Jeong-Heon Cha

**Affiliations:** 1 Department of Oral Biology, Oral Science Research Center, Yonsei University College of Dentistry, Seoul, South Korea; 2 Department of Applied Life Science, BK21 Plus Project, Yonsei University College of Dentistry, Seoul, South Korea; 3 Department of Microbiology and Immunology, Uniformed Services University of the Health Sciences, 4301 Jones Bridge Rd., Bethesda, Maryland, 20814, United States of America; 4 Departments of Cancer Biology and Medicine, Vanderbilt University, Nashville, Tennessee, 37240, United States of America; Universidad Nacional de La Plata., ARGENTINA

## Abstract

*Helicobacter pylori* genetic variation is a crucial component of colonization and persistence within the inhospitable niche of the gastric mucosa. As such, numerous *H*. *pylori* genes have been shown to vary in terms of presence and genomic location within this pathogen. Among the variable factors, the Bab family of outer membrane proteins (OMPs) has been shown to differ within subsets of strains. To better understand genetic variation among the *bab* genes and to determine whether this variation differed among isolates obtained from different geographic locations, we characterized the distribution of the Bab family members in 80 American *H*. *pylori* clinical isolates (AH) and 80 South Korean *H*. *pylori* clinical isolates (KH). Overall, we identified 23 different *bab* genotypes (19 in AH and 11 in KH), but only 5 occurred in greater than 5 isolates. Regardless of strain origin, a strain in which locus A and locus B were both occupied by a *bab* gene was the most common (85%); locus C was only occupied in those isolates that carried *bab* paralog at locus A and B. While the *babA*/*babB*/- genotype predominated in the KH (78.8%), no single genotype could account for greater than 40% in the AH collection. In addition to basic genotyping, we also identified associations between *bab* genotype and well known virulence factors *cagA* and *vacA*. Specifically, significant associations between *babA* at locus A and the *cagA* EPIYA-ABD motif (*P*<0.0001) and the *vacA* s1/i1/m1 allele (*P*<0.0001) were identified. Log-linear modeling further revealed a three-way association between *bab* carried at locus A, *vacA*, and number of OMPs from the HOM family (*P*<0.002). *En masse* this study provides a detailed characterization of the *bab* genotypes from two distinct populations. Our analysis suggests greater variability in the AH, perhaps due to adaptation to a more diverse host population. Furthermore, when considering the presence or absence of both the *bab* and *homA/B* paralogs at their given loci and the *vacA* genotype, an association was observed. Our results highlight the multifactorial nature of *H*. *pylori* mediated disease and the importance of considering how the specific combinations of *H*. *pylori* virulence genes and their multiple interactions with the host will collectively impact disease progression.

## Introduction

*Helicobacter pylori* (*H*. *pylori*) is a successful pathogen, colonizing the gastric mucosa of over 50% of the world’s population [1, 2]. This pathogen has gained notoriety for its ability to colonize the inhospitable niche of the stomach and to cause gastric diseases [1, 2]. *H*. *pylori* cause persistent, potentially lifelong infections; however, only about 20% of individuals infected will develop symptomatic infection. Although clinical manifestations occur only in a subset of infected individuals, these can be severe and include peptic ulcers and gastric cancer. Rates of *H*. *pylori* infection and gastric cancer mortality vary geographically. East Asian countries such as China, Japan, and South Korea have high rates of *H*. *pylori* infection, and these populations also have some of the highest rates of gastric cancer [3–6].

Environmental, host, and bacterial factors are known to influence *H*. *pylori*-associated disease outcome and also vary geographically [7]. Interestingly, *H*. *pylori* is a bacterial species that shows exceptionally high rates of genetic variability and intra-species diversity. Indeed, these polymorphisms have been used to determine the geographic origin of *H*. *pylori* isolates [8, 9]. Furthermore, it is highly probable that these genetic differences influence virulence. Previous studies have shown that severe disease outcomes such as gastric cancer and ulcers are associated with polymorphism in *H*. *pylori* virulence factors such as the cytotoxin-associated gene A (*cagA*) and vacuolating cytotoxin (*vacA*) [4, 6, 10–12]. In addition, disease outcome has been associated with several outer membrane proteins (OMPs). Specifically, OMPs such as AlpA and B (adherence-associated lipoprotein A and B), BabA (blood group antigen binding adhesin), HomB (*Helicobacter* outer membrane B), HopZ (*Helicobacter* outer membrane protein Z), OipA (outer membrane inflammatory protein A) and SabA (sialic acid binding adhesion) are all associated with variable disease outcomes [13–18]. In comparison to the polymorphisms within *cagA* and *vacA*, genetic variability within the OMP families is based upon presence and absence of different closely related paralogs. For example, the *bab*-family of genes is made up of three paralogs *babA*, *babB* and *babC*, which can be located at three different *H*. *pylori* chromosomal loci referred to as locus A, B and C [19–21]. Recently, we presented detailed epidemiological studies of *cagA*, *vacA*, and *homB* from a collection of 260 *H*. *pylori* clinical isolates from South Korea [11, 12, 17, 22]. Our studies showed a significant association between infection with *H*. *pylori* strains carrying the EPIYA-ABD *cagA* genotype and the development of gastric cancer [22]. Moreover, the majority of *H*. *pylori* isolates from the South Korean population encoded the most virulent toxins, CagA EPIYA-ABD motif and VacA s1/i1/m1 allele [11]. While, we found no association between the presence of *homB* and the progression to severe gastric disease [17], other groups working with isolates from different geographic regions have shown that the presence of *homB* was associated with development of peptic ulcer disease in children and young adults [23, 24] and with gastric cancer development and the presence of *cagA* [25]. The variations in these results suggest that the impact of the *homB* gene on disease is geographically dependent [17].

Our studies and others have revealed that certain polymorphisms are more prevalent within specific populations [11, 12, 17, 22, 26–29]. Thus, it is possible that the combination of virulence factor polymorphisms present within a population may influence the rate of severe disease outcome. Therefore, the polymorphism of each virulence factor must be evaluated within various populations to help understand the relationship between these genotypes and their associations with gastric diseases including cancer.

The *bab* gene family has been shown to have a positive association with gastric cancer and duodenal ulcers [6, 30–32]. BabA, one member of this family, was the first identified adhesin in *H*. *pylori* and mediates the binding of bacteria to the fucosylated blood group O antigen Lewis b (Le^b^), which is highly expressed in gastrointestinal epithelial cells [33, 34]. Similar to several *H*. *pylori* OMPs, BabA has two closely related paralogous, BabB (HopT) and BabC (HopU); however, the functions of BabB and BabC are unknown. These OMPs, particularly BabA and BabB, have nearly identical N- and C-terminal domains but a divergent middle region, suggesting that the middle variable region of BabA is most likely responsible for the specific adhesin function [19, 34–36]. Interestingly, it has been shown that BabB expression is modulated by changes in the number of cytosine-thymidine (CT) dinucleotide repeats in the 5′ region of the *babB* gene resulting in slipped-strand mispairing [37–39]. Changes in the number of CT repeats results in a frame shift; ‘IN’-frame *bab* genes are translated into a full functional Bab or ‘OUT’-of-frame *bab* genes into a premature partial Bab [37–39]. Furthermore, *bab* expression can be modulated by gene conversion between the loci, most likely by intragenomic homologous recombination [35, 40]. This gene conversion results in chimeric *bab* genes where the CT repeats of the *babB* 5′ region become associated with *babA* or *babC* subjecting these genes to phase variation. These recombination events may play an important role in regulation of adherence. For example, *babBA* chimera results in the regulation of *babA* by changes in CT repeats that lead to changes in the binding to Le^b^ on gastric epithelial cells [41].

To gain further insight into genetic diversity of *H*. *pylori* isolates within the South Korean population, we analyzed the *bab* gene family. In addition, we expanded our analysis to include clinical isolates obtained from an American hospital to allow for strain comparisons between different geographic regions.

## Materials and Methods

### Bacterial Strains and Culture Conditions

A total of 160 *H*. *pylori* clinical isolates were used in our analysis. The 80 South Korean *H*. *pylori* clinical isolates (KH) were a subset of a collection of South Korean strains previously characterized for distribution of *cagA* EPIYA polymorphism, *vacA* s/i/m polymorphism, and *homA/B* paralog [11, 12, 17, 22], while the other 80 isolates were obtained from patients at Vanderbilt University Medical Center, Nashville TN, USA. Written informed consent was received from each patient, and the protocol was approved by the Vanderbilt University and the Nashville Department of Veterans Affairs Institutional Review Board (IRB# 5190). Gastric biopsies were harvested from individuals at the Veterans Affairs Medical Center in Nashville undergoing upper endoscopy, and used for bacterial culture. Isolates from biopsies were confirmed to be *H*. *pylori* by positive urease, catalase, and oxidase tests, and typical appearance on Gram stain. These strains are referred to as American *H*. *pylori* clinical isolates (AH). For 80 AH, polymorphisms in *cagA* and *vacA*, and *homB* paralog were determined using the strategies previously developed for characterization of KH [11, 12, 17, 22]. The 160 KH and AH are described in detail in S1 Table. All *H*. *pylori* clinical isolates were cultured as previously described [22]. Briefly, bacterial isolates preserved at -80°C, were grown and expanded on antibiotic-supplemented horse blood agar plates under microaerophilic conditions created in GasPak jars with Campypak generators (Mitsubishi Gas Chemical Company, Inc, Tokyo, Japan).

### *bab* genes genotyping

**Fig 1:**
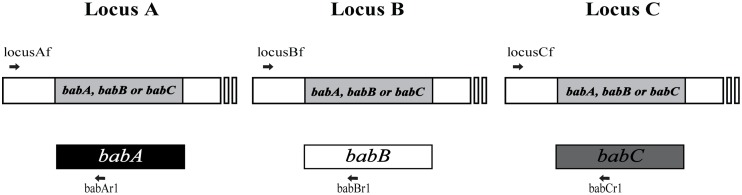
Outline of *bab* genotyping by PCR. (Top) Schematic representation of the three loci where the *bab* genes are generally detected: Locus A, B and C. The annealing positions (arrows) and names of each locus-specific forward are shown. (Bottom) Annealing positions (arrows) and names of *bab*-specific reverse primer are indicated with their respective *bab* gene: *babA* depicted by the black box, *babB* depicted by the white box, *babC* depicted by the grey box. Primers are listed in Table 1, and a full explanation of the genotyping scheme can be found in the Materials and Methods.

Chromosomal DNA was extracted from *H*. *pylori* strains G27, J99, and 26695 and from the 160 clinical isolates using the G-spin total DNA extraction kit (Intron Biotechnology, inc., Seoul, South Korea). Primers used for genotyping and sequencing *bab* genes in this study were based on the genome sequences of *H*. *pylori* strains G27, J99 and 26695 (GenBank accession numbers CP001173, AE001439, and CP003904, respectively) (Table 1). PCRs using a locus-specific forward primer and *bab*-specific reverse primer (babAr1, babBr1 or babCr1) were carried out to investigate the presence or absence of *babA*, *babB* or *babC*, respectively (Fig 1) [20, 42]. If all three PCRs per locus were negative, the locus was considered empty. Sequencing of amplicons was used to identify *bab* chimeras and whether the *bab* genes were ‘IN’-frame or ‘OUT’-of-frame according to the number of CT repeats. In addition, to assess the entire *babC* gene (approximately 2200 bp), PCR amplicons of locusAf/babCr1 and babCf1/locusAr were sequenced using primers babCf1, babCf2, babCf3, babCr1, babCr2, locusAf, and locusAr and the resulting overlapping DNA fragments were aligned. Sanger dideoxy sequencing was performed at Cosmo Genetech Co., Ltd. (Seoul, South Korea) and Geno Tech Corp (Seoul, South Korea), and the resulting DNA sequences were analyzed using Vector NTI version 9.1 (Invitrogen, Carlsbad, CA,USA) and Sequencher 5.1 (Gene Codes Corp., Ann Arbor, MI, USA).

**Table 1:** Primer sequences for analysis of *H*. *pylori bab* genes.

Primer name	Primer sequence (5’→3’)	Reference
locusAf	TTTTGAGCCGGTGGATATATTAG	HypDF1 [42] ^d^
locusBf	CTTTAATCCCCTACATTGTGGA	S18F1 [42] ^d^
locusCf ^a^	ACCCTAGTGGGCATGTGGTA	Hp1-AS [20] ^d^
locusAr ^b^	GGAAATGCGCACACGAGGG	this study
babAr1 ^b^	TTTGCCGTCTATGGTTTGG	BabAR1 [42] ^d^
babAr2 ^b^	GAAAGGATGTGTTTTTTCATG	this study
babBr1 ^b^	TCGCTTGTTTTAAAAGCTCTTGA	BabBR1 [42] ^d^
babBr2 ^b^	CTGCCAGGACCACAAGCAGT	this study
babCf1 ^b^	GCGGCAAACATCATGCAAGTC	this study
babCr1 ^b^	GACTTGCATGATGTTTGCCGC	this study
babCf2 ^b^	CGGGTTTGCTCAAAGAAAAAAYC ^c^	this study
babCr2 ^b^	GRTTTTTTCTTTGAGCAAACCCG ^c^	this study
babCf3 ^b^	AAAGAATAACCCCTATAGCC	this study

^*a*^ One nucleotide in the primer was modified from the indicated primer in reference.

^*b*^ Primer was used for sequencing.

^*c*^ Underlined Y indicates C or T, and underlined R indicates G or A.

^*d*^ Primer name was used in the indicated reference.

### Phylogenetic Analysis

Phylogenetic trees were constructed using Phylip v3.695 (Phylogeny Inference Package) [43]. First, an input file was generated based on gene variation at seven distinct loci: *cagA* (EPIYA type), *vacA* (s/i/m type), *hom* loci A/B (*hom* type) and *bab* loci A/B/C (*bab* type). Each locus was assigned a discrete numerical character corresponding to its type. Consequently, each clinical isolate was distinguished by a seven-number code representing its genotype. For example, strain G27 has the following genotype: *cagA* EPIYA (ABCC), *vacA* (s1/i1/m1), *hom* locus A (*homB*), *hom* locus B (empty), *bab* locus A (*babC*), *bab* locus B (*babB*), and *bab* locus C (*babA*). This genotype corresponds to the number 2020321 in the input file. All resulting codes were utilized to generate the strain input files used in subsequent analyses (S3 Table).

We began constructing the trees via the SEQBOOT program to generate multiple data sets. With this program we used a combination of bootstrapping and character permutation to generate 100 data sets. These data sets were then used as the input file in the PARS program, a general parsimony program that carries out the Wagner parsimony method and allows for up to 8 discrete character states and the use of ‘?’ for states that are unknown. PARS was run using the default settings with the following exceptions: species order was randomized using a random number seed of 5, with 10 replicates and the trees were constructed from the 100 data sets generated in SEQBOOT. PARS searches for the best fit tree, saving the 100 best options. As a final step, we used the PARS output trees in the CONSENSE program to generate a majority-rules consensus tree. In doing this, the branch lengths of our final tree represent the number of times a given branching event occurred in the 100 trees generate by PARS. FigTree v1.4.2 is a tree editing program [44].

### Statistical Analysis

Statistical analysis was conducted as previously described [12]. Briefly, two-way associations between *bab* genotype, *hom* genotype, *cagA* EPIYA polymorphisms, *vacA* s/i/m polymorphisms, and disease state were analyzed using the Fisher’s exact test. Log linear modeling was used to assess higher order associations that were significant at a 5% level. Using categorical values to represent our data sets, we fit a saturated model. We then applied a backward selection algorithm that eliminates the least significant associations at each step and then reforms the model to look for associations. Data analysis was conducted using SPSS version 22 software (SPSS Inc., Chicago, IL, USA) or SAS version 9.3 software (SAS Institute Inc., Cary, NC, USA).

### Nucleotide Sequence Accession Numbers

The sequences for *bab* genotypes in American and South Korean strains were deposited in the NCBI GenBank database with accession numbers KP339308 to KP339491.

## Results

### Sample Population

Complete demographic data was available for all 80 AH (S1A and S2A Tables). For the American population, the mean patient age was 57 years, with an age range of 27–84 years. 20.0% (16 patients) of the population was black, and 80.0% (64 patients) was white. Finally, 79 of the patients were male; there was one white female patient, age 50. Of these clinical isolates, 7.5% were from patients with cancer/pre-malignant lesions (2.5% with gastric carcinoma and 5.0% with Barrett’s Esophagus), 43.8% were from patients with peptic ulcer disease (31.3% with duodenal ulcers and 12.5% with gastric ulcers), 32.5% were from patients with gastritis, and 16.3% were from patients with esophagitis. Within the South Korean population, age and gender were missing for 2 individuals. Of the remaining 78, the mean age was 48 years, ranging from 20–86 years of age. The South Korean population had a more even distribution between males and females, with 48.7% (38 patients) female and 51.3% (40 patients) male. Within the South Korean female patient group, the mean age was 51, with an age range of 21–86 years. Within the South Korean male patient group, the mean age was 46, with an age range of 20–76 years. Of the 80 KH samples analyzed, 18.8% were from patients with gastric cancer, 33.8% were from patients with peptic ulcer disease (32.5% with duodenal ulcers and 1.3% with gastric ulcers), and 47.5% were from patients with gastritis.

### *bab* genes genotyping

**Fig 2:**
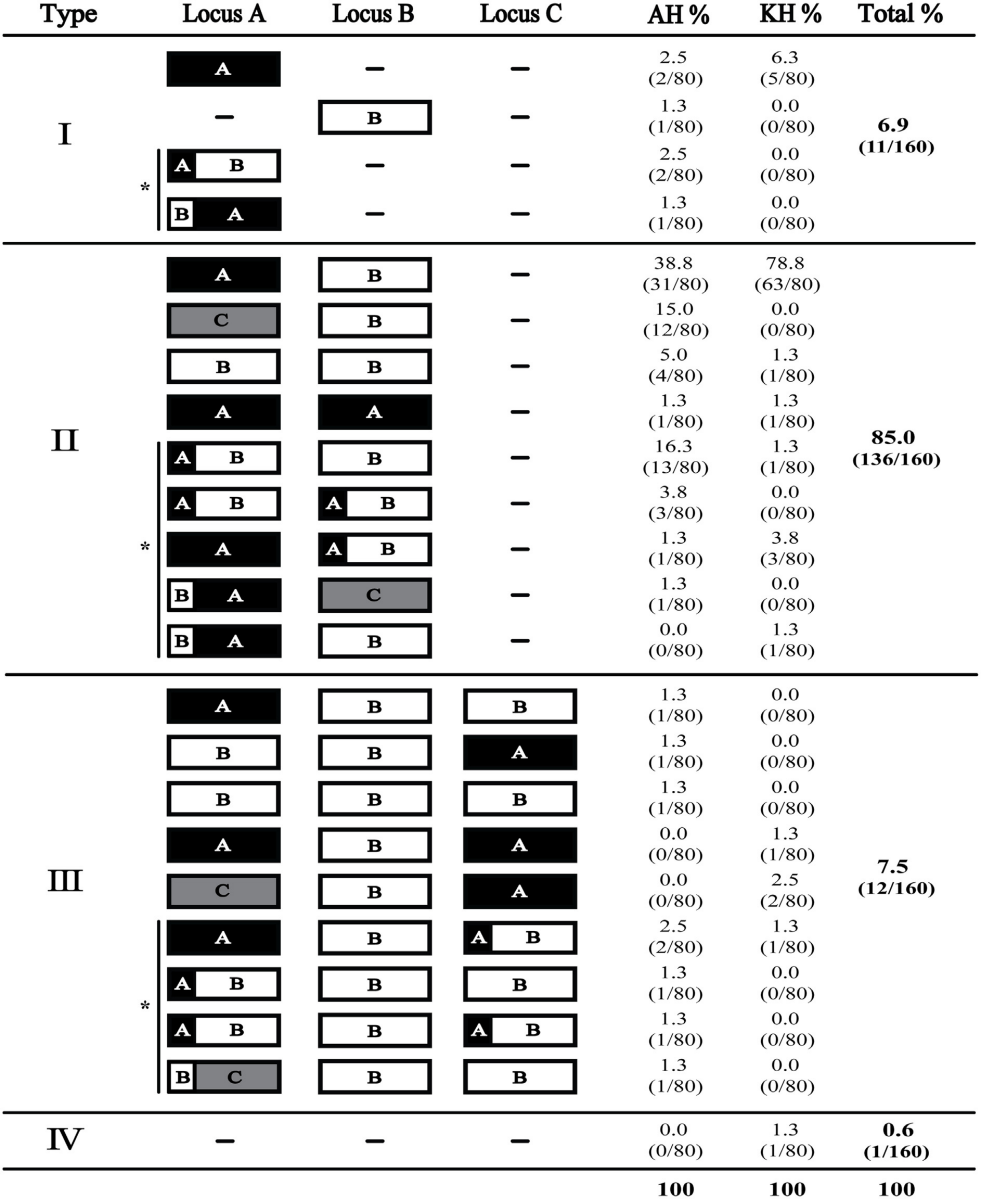
Distribution of the *bab* genotypes in AH and KH (n = 160). Genotypes of one, two, three, or none *bab* genes are grouped into I, II, III, and IV, respectively. These classifications were made on the basis of number of *bab* genes present at locus A, B, and C. A black, white, or grey box indicated *babA*, *babB*, and *babC*, respectively. ‘-’ indicates the absence of *bab* gene at the designated loci. ‘AH%’ and ‘KH%’ indicate the frequency of each individual genotype within the specified population (n = 80) and ‘Total%’ shows the percent of the respective genotype in the overall population (n = 160). Bracketed numbers indicate the number of isolates possessing each respective genotype out of the population examined. ‘_*_’ indicates genotypes containing chimeric form(s).

The distribution of identified *bab* genes at locus A, B and C is shown in Fig 2. Genotypic analysis revealed that in addition to *babA*, *babB* and *babC*, three types of chimeric *bab* genes were also present: *babAB*, *babBA* and *babBC*. A total of 23 different *bab* genotypes, including a single strain where *bab* was absent from the three known loci (-/-/-), were identified within the 160 isolates chosen for this study. While 6.9% of strains (11/160) carried a single *bab* gene, the majority of the strains (85.0%, 136/160) carried two *bab* genes, one at locus A and one at locus B (Fig 2). Of note, locus C was only occupied if both other loci also carried a *bab* gene (Fig 2). In the KH, 11 of the 23 genotypes were identified, and isolates carrying two *bab* genes were most common, accounting for 87.5% of the isolates. The genotype *babA*/*babB*/-, position 1, 2, and 3 correspond to locus A, B and C, respectively, occurred in the 78.8% of the isolates (Fig 2). In comparison, there were many more *bab* genotypes within the AH. In fact, 19 of the 23 genotypes were identified. Twelve genotypes were unique to the AH while 4 were unique to the KH. Similar to the KH, isolates carrying two *bab* genes were most common among AH, accounting for 82.5% of the isolates; however, while the most common genotype of *babA*/*babB*/- occurred in only 38.8%, genotypes of *babAB*/*babB*/- (16.3%) and *babC*/*babB*/*-* (15.0%) showed a higher frequency in this population (Fig 2). Whereas locus A of KH was occupied by *babA* in greater than 90% of isolates (74/80), most of the diversity within the *bab* genotypes for the AH was driven by locus A, which was significantly more variable (*P*<0.0001) than seen in KH. Indeed, *babA* occurred at locus A only 47.5% of the time (S1 Fig). The next most frequent occupants of locus A were chimeric *bab* genes (28.8%), followed by *babC* (15.0%) and *babB* (7.5%).

In total, we identified 3 chimeric *bab* genes, *babAB*, *babBA* and *babBC* that were the result of gene conversion. This gene conversion likely occurs in the 5′region (~60 to 240 bp from the initiation codon) and in the 3′region (~1200 to 2100 bp) but this later event is hard to detect due to the homology in this region. It can be speculated that the characteristic of the chimera would follow that of the second *bab* gene, which would constitute the majority of the chimeric gene. *bab* chimeric forms were relatively common in the AH group, occurring at one or more loci in 26/80 (32.5%) of isolates versus 6/80 (7.5%) in the KH group (Fig 2 and S2 Table). The most common chimeric gene was *babAB*, which accounted for 88.9% of chimeric genes; 90.0% of chimeras in AH (27/30) and 83.3% in KH (5/6) (Fig 2 and S2 Table). Within the *babB* coding sequence there are variable CT repeats approximately 56 bp downstream from the initiation codon that are responsible for phase variation. In the *babAB* chimera, this region is displaced with the corresponding part of *babA* [41], resulting in loss of phase variable *babB*. Although it appeared much less frequently, *babBA* and *babBC* chimeras were also present (Fig 2 and S1 Fig). In these instances, CT repeats from the 5′region of *babB* were introduced to *babA* and *babC*, respectively, resulting in phase variable *babA* and *babC*.

Eighty-two *bab* genes from the AH group and 72 *bab* genes from the KH group contained a run of CT repeats (5–14 CT repeats) (Table 2). In all cases, the CT repeats were associated with the 5′ region of *babB* genes; therefore, *babBC* and *babBA* also carry CT repeats. CT repeats were found at all three loci within the AH, with the majority (84.2%) occurring at locus B (Table 2). Similarly, in the KH group a majority of the *bab* genes containing CT repeats were found at locus B (97.2%) (Table 2). Interestingly, when comparing locus B in the AH group to the KH, significantly more *babB* genes were ‘IN’-frame in the KH group than in the AH (*P* = 0.0341, data not shown). Although not significant, the trend was similar for all *babB* genes regardless of loci, where more *babB* genes were ‘OUT’-of-frame (54.9%) than ‘IN’-frame (45.1%) in the AH group (S2 Fig). The opposite trend was observed in the KH group; *babB* genes tended to be ‘IN’-frame (59.7%) as compared to ‘OUT’-of-frame (40.3%) (S2 Fig).

**Table 2:** ‘IN’-frame or ‘OUT’-of-frame status deduced by the number of CT dinucleotide repeats in the *babB* coding region.

Group-Locus	Frame Status	Pattern of CT repeats	Sequence of CT-repeats region	Number Identified*
**AH-A (9)**	**IN**	**8**	**ATGAAAAAAACCCTTTTACTCTCTCTCTCTCTCTCGTTTTTG**	**4**
	**IN**	**11**	**ATGAAAAAAACCCTCCTACTCTCTCTCTCTCTCTCTCTCTCGTTTTTG**	**1**
	**OUT**	**10**	**ATGAAAAAAACCCTTTTACTCTCTCTCTCTCTCTCTCTCGTTTTTG**	**2**
	**OUT**	**9**	**ATGAAAAAAACCCTTTTACTCTCTCTCTCTCTCTCTCGTTTTTG**	**1**
	**OUT**	**7**	**ATGAAAAAAACCCTTTTACTCTCTCTCTCTCTCGTTTTTG**	**1**
**AH-B (69)**	**IN**	**8**	**ATGAAAAAAACCCTTTTACTCTCTCTCTCTCTCTCGTTTTTG**	**17**
	**IN**	**11**	**ATGAAAAAAACCCTTTTACTCTCTCTCTCTCTCTCTCTCTCGTTTTTG**	**10**
	**IN**	**14**	**ATGAAAAAAACCCTTTTACTCTCTCTCTCTCTCTCTCTCTCTCTCTCGTTTTTG**	**2**
	**OUT**	**9**	**ATGAAAAAAACCCTTTTACTCTCTCTCTCTCTCTCTCGTTTTTG**	**15**
	**OUT**	**10**	**ATGAAAAAAACCCTTTTACTCTCTCTCTCTCTCTCTCTCGTTTTTG**	**10**
	**OUT**	**7**	**ATGAAAAAAACCCTTTTACTCTCTCTCTCTCTCGTTTTTG**	**8**
	**OUT**	**6**	**ATGAAAAAAACCCTTTTACTCTCTCTCTCTCGTTTTTG**	**4**
	**OUT**	**12**	**ATGAAAAAAACCCTTTTACTCTCTCTCTCTCTCTCTCTCTCTCGTTTTTG**	**2**
	**OUT**	**13**	**ATGAAAAAAACCCTTTTA** **CTCTCTCTCTCTCTCTCTCTCTCTCT** **CGTTTTTG**	**1**
**AH-C (4)**	**IN**	**8**	**ATGAAAAAAACCCTTTTACTCTCTCTCTCTCTCTCGTTTTTG**	**3**
	**OUT**	**10**	**ATGAAAAAAACCCTTTTACTCTCTCTCTCTCTCTCTCTCGTTTTTG**	**1**
**KH-A (2)**	**IN**	**8**	**ATGAAAAAAACCCTTTTACTCTCTCTCTCTCTCTCGTTTTTG**	**1**
	**OUT**	**10**	**ATGAAAAAAACCCTTTTACTCTCTCTCTCTCTCTCTCTCGTTTTTG**	**1**
**KH-B (70)**	**IN**	**8**	**ATGAAAAAAACCCTTTTACTCTCTCTCTCTCTCTCGTTTTTG**	**31**
	**IN**	**11**	**ATGAAAAAAACCCTTTTACTCTCTCTCTCTCTCTCTCTCTCGCTTTTG**	**7**
	**IN**	**5**	**ATGAAAAAAACCCTTTTACTCTCTCTCTCGTTTTTG**	**4**
	**OUT**	**10**	**ATGAAAAAAATCCTTTTACTCTCTCTCTCTCTCTCTCTCGTTTTTG**	**10**
	**OUT**	**9**	**ATGAAAAAACCCCTTTTTCTCTCTCTCTCTCTCTCTCGTTTTTG**	**7**
	**OUT**	**7**	**ATGAAAAAAACCCTTTTACTCTCTCTCTCTCTCGTTTTTG**	**6**
	**OUT**	**TT+6**	**ATGAAAAAAACCCTTTTATTCTCTCTCTCTCTCGTTTTTG**	**3**
	**OUT**	**TT+8**	**ATGAAAAAAACCCTTTTATTCTCTCTCTCTCTCTCTCGTTTTTG**	**1**
	**OUT**	**12**	**ATGAAAAAAACCCTTTTACTCTCTCTCTCTCTCTCTCTCTCTCGTTTTTA**	**1**

* This number corresponds to the number of times the specific CT-repeat pattern was identified at the given locus.

() The bracketed number indicates the number of times a *bab* gene carrying CT repeats was identified.

**Fig 3:**
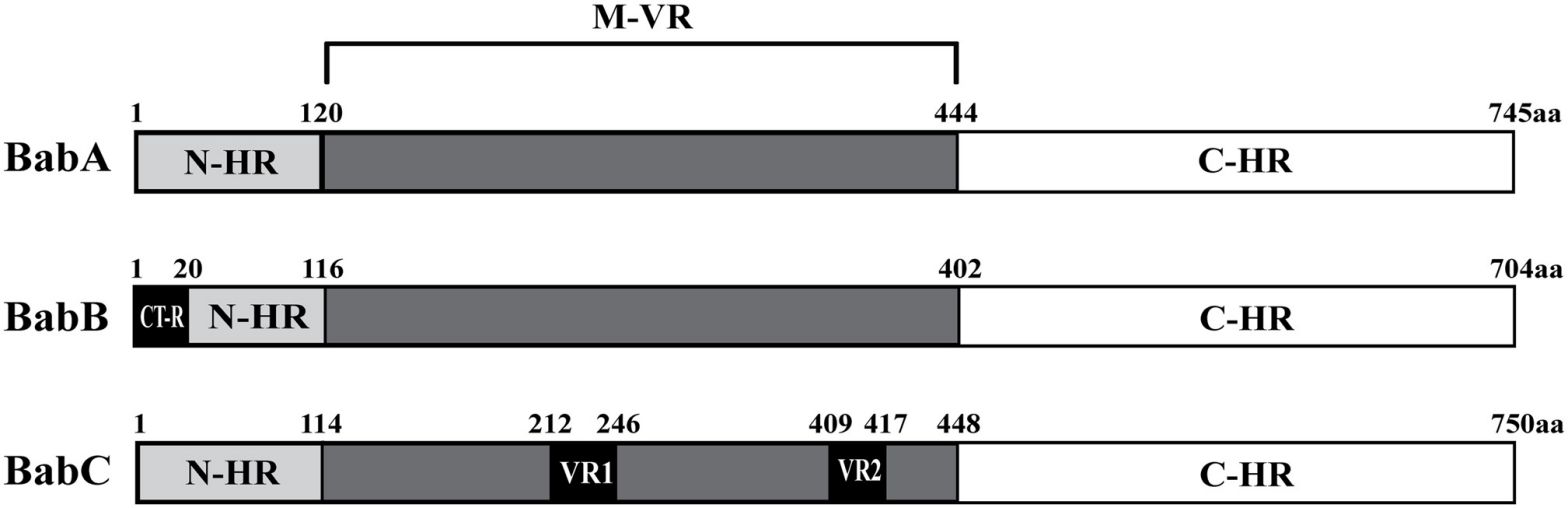
Schematic comparison of BabA, BabB, and BabC. The representative structures of BabA and BabB are based on the J99 amino acid sequence, whereas the structure of BabC is based on the consensus sequence defined in this study. The N-HR indicates N-terminal homology region, M-VR indicates middle variable region, which is characterized by sequence difference among the three Bab proteins. Note that the M-VR is conserved for each of the Bab proteins. The C-HR indicates C-terminal homologous region that shows >90% identity. The CT-R present at the N-terminus of BabB refers to the CT repeat region. VR-1 and VR-2 in BabC indicate regions of variability among the BabC amino acid sequences analyzed in this study (n = 15).

Since very little is known about *babC*, we took this opportunity to sequence 15 *babC* genes from our collection and compare the deduced BabCs. As shown in S3 Fig we defined a BabC consensus sequence by comparing 14 BabCs and one BabBC from this study along with the BabC from G27. According to the *babC* sequences, we identified only one chimeric *babBC* gene (AH-J68) and no *babAC* gene. Furthermore, we found that the N-terminus of BabC was most similar to that of BabA, and the C-terminus of BabC was homologous to that of both BabA and BabB (Fig 3). The amino acid sequence of BabC contained two variable regions, VR1 spanning from amino acid 212 to 246 and VR2 spanning from amino acid 409 to 417. The BabC sequences, including VR1 and VR2, shared over 70% identities (Fig 3 and S3 Fig).

To further understand the *bab* genotypes in these populations, we next sought to determine if carrying a specific *bab* gene at one locus was associated with the presence of *bab* genes at the other loci. Given the difference in *bab* genotypes between the two populations, we first examined each population separately. A significant association was found in the KH group between the genotype at locus A and locus C (*P* = 0.0027) (Table 3). Conversely, in the AH group a significant association was found between locus A and locus B (*P* = 0.0028). When KH and AH were combined, there was also a significant association between locus A and locus B (*P* = 0.0131) (Table 3). Of note, in the AH/KH-combined analysis *babC* was more likely to occur at locus A when *babB* at locus B was ‘OUT’-of-frame (78.6%, 11/14). Furthermore, if BabB was being expressed from locus A, then *babB* at locus B was more likely to be ‘OUT’-of-frame (60.0%, 15/25). Yet interestingly, if *babA* occupied locus A, then *babB* at locus B was more likely to be ‘IN’-frame (58.9%, 66/112).

**Table 3:** Significant two-way comparisons of *bab* genotype and other factors in both populations^a^.

*bab* genotype comparison	*P* value^b^ for distribution within		
	KH	AH	Combined^c^
*babA*,*B*,C or empty at Locus A vs *babA*,*B*,*C* or empty at Locus B	0.1046	**0.0028**	**0.0131**
*babA*,*B*,C or empty at Locus A vs Full or empty *bab* at Locus C	**0.0027**	0.4111	0.4500
*babA*,*B*,C or empty at Locus A vs one or two *hom* loci occupied	**0.0167**	**<0.0001**	**0.0029**
*babB* ‘IN’ or Other at Locus B vs one or two *hom* loci occupied	**0.0210**	1.0000	0.6070
Full or empty *bab* at Locus C vs one or two *hom* loci occupied	1.0000	**0.0390**	0.1750
*babA*,*B*,C or empty at Locus A vs *homA or homB*	0.6390	**<0.0001**	**<0.0001**
*babA*,*B*,*C* or empty at Locus B vs *homA* or *homB*	0.7340	**0.0160**	**0.0260**
*babA*,*B*,C or empty at Locus A vs v*acA* s1 or *vacA* s2	1.0000	**<0.0001**	**<0.0001**
*babA*,*B*,C or empty at Locus A vs v*acA* i1 or *vacA* i2	0.9999	**<0.0001**	**<0.0001**
*babA*,*B*,C or empty at Locus A vs v*acA* m1 or *vacA* m2	0.9999	**<0.0001**	**<0.0001**
*babA*,*B*,C or empty at Locus A vs *cagA* EPIYA-ABD or Other	0.5010	N/A*	**<0.0001**
*babA*,*B*,*C* or empty at Locus B vs Cancer/Gastric ulcer or Duodenal ulcer/Gastritis	**0.0101**	0.828^#^	0.483^#^
*babB* or other at Locus B vs Cancer or Gastric ulcer or Duodenal ulcer or Gastritis	**0.0012**	0.806^#^	0.447^#^

^*a*^ For simplicity, Table 3 only contains associations for which a statistically significant association was found in at least one grouping; however, an exhaustive analysis was conducted on numerous other permutations of the data.

^*b*^ Statistically significant *P* values are in boldface type.

^*c*^ All isolates (n = 160) analyzed as a single group

*No ABD in the AH

^#^Also includes a category for Esophogitis/Barrett's Esophagus.

### Associations between *bab* and *hom* genes

Given the associations among *bab* loci, we next assessed whether *bab* genotype was associated with other outer membrane proteins. We have previously looked at the association between *homB* and disease in the South Korean population [17]. *hom* paralog (*homA* and *homB*) from the Hom protein family, which shares over 90% identity, are known to occupy two possible loci (also called locus A and B) [45]. Therefore, we chose to evaluate any association between the *bab* genes, and *homA* and *homB*. Once again, given the differences in the two populations, each was analyzed individually as well as combined. There were no significant associations when comparing strains for presence of *homA* versus *homB*. Given that *homA* and *homB* are homologous and share two conserved loci, locus A and locus B, we also considered *hom* gene number in which we looked at whether or not both *hom* locus A and locus B were occupied regardless of if it was *homA* or *homB*. We found a significant association with the *bab* paralog present at locus A and number of *hom* loci occupied. This association was seen in both the AH and KH groups (*P*<0.0001 and *P* = 0.0167, respectively) (Table 3). In the AH group, presence of a *babB* at locus A was more often associated with a single *hom* gene (61.5%, S1A and S2A Tables). Conversely, in the KH *babA* at locus A occurred most often with a single *hom* (97.3%, S1B and S2B Tables). When the groups were combined, the association between number of *hom* loci occupied and the *bab* gene at locus A remained significant (*P* = 0.0029) (Table 3). One factor that stands out when the groups were combined was that although *babC* was rare (n = 14), it occurred only in isolates with a single *hom* gene. Also of note in the AH group, in strains where locus C was occupied there was a significant difference between carrying a single *hom* gene versus two *hom* genes (*P* = 0.039). Although the finding wasn’t significant in the KH group, the same trend was seen; locus C was only occupied in strains with a single *hom* gene (Table 3 and data not shown).

### Associations between *bab*, *cagA*, and *vacA*

It has been well established that particular *cagA* and *vacA* polymorphisms are associated with more severe disease outcomes [12, 22, 27, 46]. Due to their association with disease outcome, we next assessed whether particular *bab* genotypes were found in association with known *cagA* or *vacA* polymorphisms. There were no significant associations between *cagA* and *bab* when the groups were looked at separately; however, when considering the clinical isolates as a whole, there was a significant association between the *bab* gene at locus A and the *cagA* EPIYA polymorphism (*P*<0.0001) (Table 3). The *cagA* EPIYA-ABD polymorphism occurred more frequently in strains that carry a *babA* at locus A (92.9%, 65/70), whereas strains carrying a *babB* or *babC* at locus A were most likely to have a non EPIYA-ABD (93.1%, 27/29 and 85.7%, 12/14, respectively) (S1 and S2 Tables). Similar to *cagA*, *vacA* associations were only apparent when KH and AH were combined. When we analyzed all 160 isolates, there were significant associations between *bab* at locus A and the s, i, and m regions of *vacA* (*P*<0.0001 for each) (Table 3). Further analysis of the associations between the *vacA* polymorphic regions and *bab* at locus A uncovered a three-way association between *bab* at locus A, the *vacA* s, i, m regions, and occupation of a single or both *hom* loci (Table 4). If we first consider strains carrying *vacA* s1 polymorphisms, these strains were most likely to harbor a *babA* at locus A. Interestingly, *babB* occurs almost equally with the s1 and s2 *vacA* polymorphisms; however, 100.0% of strains containing *babB* at locus A and s2 carried a single *hom* gene compared to strains carrying s1, which were more likely to carry two *hom* genes. The association between s2/*babB*/single *hom* and s1/*babB*/two *hom* genes were both significant (*P* = 0.00062 and *P* = 0.01846, respectively, data not shown). The i and m regions of *vacA* clustered together in that i1 compared to i2 and m1 compared to m2 resulted in the same distributions. *babA* at locus A was more frequently associated with i1 and m1 as compared to *babC*, which was much more commonly associated with i2 and m2. Once again, *babB* appeared to be found in relatively equal frequencies with i1m1 and i2m2; however, when we analyzed the *vacA* genotype of strains carrying *babB* at locus A, the strains with only an i2m2 *vacA* type were more likely to have a single *hom* gene (87.5%, 14/16, *P* = 0.00744) and strains with an i1m1 *vacA* classification were more likely to carry two *hom* genes (76.9%, 10/13, *P* = 0.00166) (data not shown).

**Table 4:** Three-way comparisons of *bab* genotype and other factors in both populations.

*bab* genotype comparison	*P* value^a^ for distribution within		
	KH	AH	Combined^b^
*babA*, *B* or *C* at Locus A vs *vacA* i1/i2 vs one or two *hom* loci occupied	N/A	0.097	**0.001**
*babA*, *B* or *C* at Locus A vs *vacA* s1/s2 vs one or two *hom* loci occupied	N/A	0.173	**0.002**
*babA*, *B* or *C* at Locus A vs *vacA* m1/m2 vs one or two *hom* loci occupied	N/A	0.882	**0.001**
*babA*, *B* or *C* at Locus A vs *vacA* s1i1m1/other vs one or two *hom* loci occupied	1.000	N/A^#^	N/A^#^
*babA*, *B* or *C* at Locus A vs *cagA* (AB &Other^$^/ABCs/ABD*)* vs *vacA* m1/m2	N/A	1.0000	0.158
*babA*, *B* or *C* at Locus A vs *cagA* (AB &Other^$^/ABCs/ABD) vs *vacA* s1/s2	N/A	0.963	0.881
*babA*, *B* or *C* at Locus A vs *cagA* (AB &Other^$^/ABCs/ABD) vs *vacA* i1/i2	N/A	0.916	0.158
*babA*, *B* or *C* at Locus A vs *cagA* (Other/ABD) vs *vacA* s1i1m1/other	1.0000	N/A^#^	N/A^#^
*babA*, *B* or *C* at Locus A vs *cagA* (AB &Other^$^/ABCs/ABD) vs one or two *hom* loci occupied	0.976	1.000	**0.044**
*babA*, *B* or *C* at Locus B vs one or two *hom* loci occupied vs *vacA* s1/s2	N/A*	0.823	0.061

^*a*^ Statistically significant *P* values are in boldface type.

^*b*^ All isolates (n = 160) analyzed as a single group.

^#^These comparison were only done in KH; for AH and combined we looked at i, s, and m regions of *vacA* separately, which wasn’t feasible with KH since was overwhelmingly s1/i1/m1.

*All KH strains are *vacA* s1.

^$^AB&Other refers to any *cagA* EPIYA motif that is not ABC_(1–4)_, or ABD.

### Associations between *bab* genotype and disease

Given the association between the virulence factors, *cagA* and *vacA* and the *bab* genes, particularly at locus A, we asked if there was an association between *bab* genotype and disease. The only significant association between *bab* and disease was seen in the KH group. In this group, there was a significant association between the *bab* genes present at locus B and disease outcome (*P* = 0.0101) (Table 3). Specifically, this association appeared to be driven by the fact that isolates that lacked a *bab* gene at locus B were more likely (66.7%) to come from individuals suffering from more severe disease outcome (gastric cancer and gastric ulcers) than from individuals suffering from gastritis and duodenal ulcers (33.3%). It was also interesting to note, although the finding was not significant (*P* = 0.176), that individuals with cancer (gastric carcinoma) or pre-malignant lesions (Barrett’s Esophagus) were more likely to have a *babB* gene that was ‘IN’-frame’ (82.4%, 14/17) compared to ‘OUT’-of-frame’ (17.6%, 3/17) (data not shown).

### Phylogenetic Analysis of AH and KH

**Fig 4:**
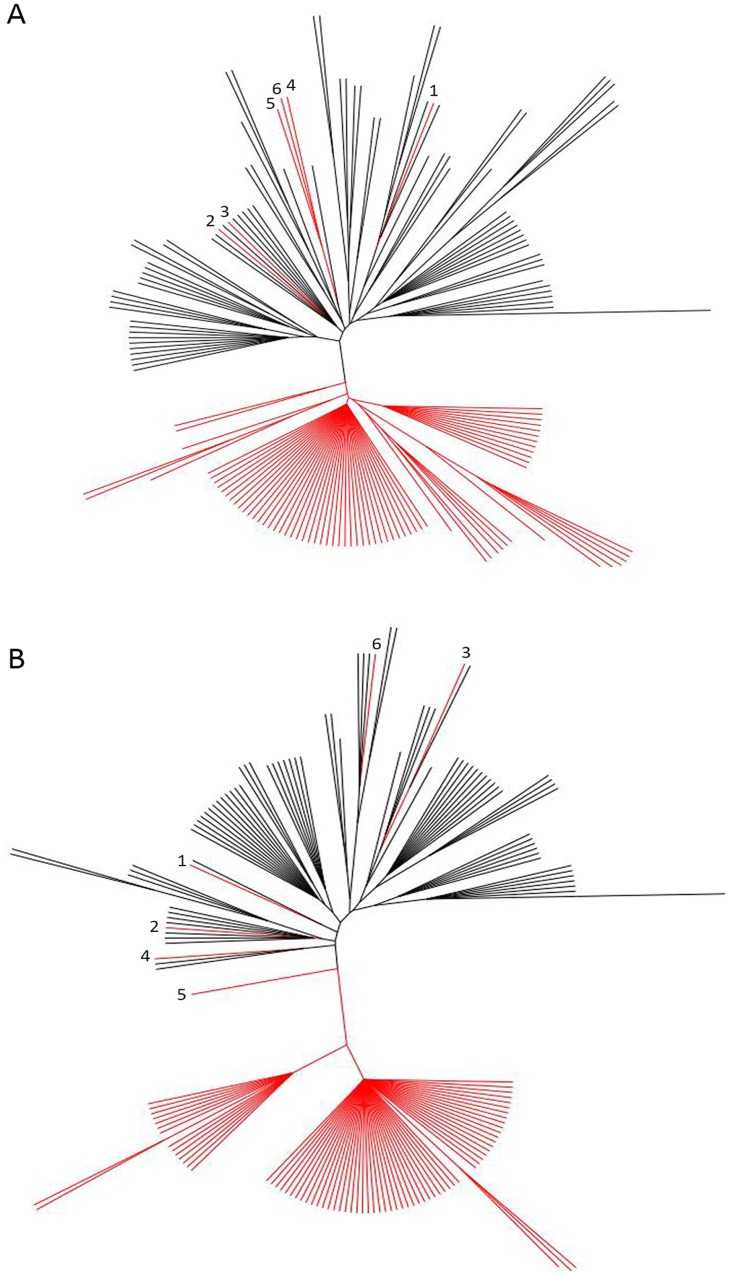
Phylogenetic analysis of AH and KH. (A) An unrooted consensus tree based on *cagA* EPIYA type, *vacA* s/i/m type, *homA/B* genotype, and *bab* genotype. (B) An unrooted consensus tree based on *homA/B* and *bab* genotypes only. Trees were created in PHYLIP with Wagner parsimony followed by the majority-rules consensus method. KH branches/nodes are red and AH branches/nodes are black. The six KH grouped with AH are identified by numbers 1–6 in both trees and correspond to the following strains:(1) K47, (2) K78, (3) K262, (4) K57, (5) K24, and (6) K197.

To complement our statistical analysis, we also conducted a phylogenetic analysis based on gene variation at seven loci: *cagA* (EPIYA type), *vacA* (s/i/m type), *hom* locus A and B (*hom* type), and *bab* locus A, B, and C (*bab* type). As shown in Fig 4A, using these parameters led to a distinct geographical separation between the KH and AH. Only, 6 KH (7.5%) cluster with the AH: K47, K78, K262, K57, K24, and K197. Interestingly, while most of these strains have the predominant KH *cagA* ABD EPIYA motif and *vacA* s1/i1/m1 type, their *bab* and *hom* genotypes more closely resembled the AH. Given this observation, we next constructed a separate consensus tree using only the *hom* and *bab* genes (Fig 4B). Remarkably, this tree was very similar to the tree generated using all 7 loci; once again, the majority of the KH and AH segregated, and the same 6 KH grouped more closely with the AH. Closer inspection of the OMP genotypes revealed that the *hom* genotypes of the 6 KH that group among the AH, deviate from the single *hom* at locus B genotype that predominates in the KH.

## Discussion

The *bab* loci have been investigated in detail in several human populations as well as in long term animal colonization studies [21, 39, 47–49]; however, in this study we not only asked what the *bab* genotypes were in South Korean and American populations, but also which *bab* genes were phase variable, which of the phase variable *bab* genes were ‘IN’-frame or ‘OUT’-of-frame, and how the *bab* genotypes were associated with other genetic markers. Furthermore, we sought to compare these findings between two distinct populations. The KH came from a population with high levels of *H*. *pylori* infection and a large burden of stomach cancer, while the AH came from a population known to have lower levels of infection and stomach cancer. Although higher rates of *H*. *pylori* infection in the KH group can partially explain the increased rates of stomach cancer, it is also possible that genetic differences between the *H*. *pylori* circulating in the South Korean compared to the strains circulating in the United States may also play a role. For our study we began by genotyping the three *bab* loci in 80 clinical isolates obtained from South Korea and 80 clinical isolates from the United States. Overall, we identified 23 different *bab* genotypes. Of these 23, 19 were found in the AH and 11 in the KH group (Fig 2). Interestingly, although there are a thousand potential *bab* genotypes, our work and previous studies have shown that not all combinations are observed [21, 42, 47, 50]. For example, consistent with previous studies [21, 42, 50], we found that carrying two *bab* genes at locus A and locus B was the most common genotype. In the KH group, close to 80% of isolates showed *babA*/*babB*/- genotype. Conversely, even though the AH group did not have one genotype that was found in greater than 40% of the population, the majority of isolates still carried two *bab* genes: *babA*/*babB*/-, *babAB*/*babB*/-, or *bab*C/*babB*/-. Our data suggest that while there are a large number of possible combinations of the *bab* genes at different loci, certain genotypes predominate and the others are rare. Although we identified 23 genotypes, only 5 genotypes were found in at least 5 of the isolates, and 4 genotypes were found only within the KH group while 12 genotypes were exclusive to the AH group. Also of note, in both the KH and AH collections, locus A was most frequently occupied by *babA*, and locus B by *babB*. Furthermore, locus C was only occupied when locus A and B carried a *bab* gene (Fig 2 and S1 Fig). Taken together, these data show that isolates carrying two *bab* genes are most common regardless of geographic origin of the strain. Based upon these observations, one might speculate that in an ancestral strain locus A was occupied by *babA* and locus B by *babB*, and as *H*. *pylori* adapted, gene conversion events have resulted in varying *bab* genotypes. Since the *bab* genes encode OMPs, and since BabA is known to act as an adhesin, it is likely that differences in the *bab* genotype affect how the bacteria interacts with the host. Therefore, perhaps the genotypes that appear to occur in only 1 or 2 individuals reflect adaptation of *H*. *pylori* to an individual host, or across gastric regions within a host, and also within an individual over time [42].

In addition to *bab* locus genotyping, we also examined which *bab* genes were subject to phase variation. As previously shown, CT repeats associated with the 5′ region of *babB* can result in slipped-strand mispairing leading to a premature stop in translation [37–39]. In the KH group, *babB* at locus B was significantly more likely to be ‘IN’ frame’ compared to the AH which may reflect a difference in the host populations since *H*. *pylori* likely uses phase variation as a response to host changes. Probably through gene conversion, CT repeats were also identified in association with *babA* and *babC* at locus A. These data suggest that gene conversion occurred in these strains and resulted in phase variable *babA* and *babC*, imparting *H*. *pylori* with additional means of regulation over these genes. Interestingly, the most common gene conversion of *babAB* among 3 types of chimeric *bab* genes deprived *babB* of the regulation of phase variation.

Evidence from this study and others suggest that varying the *bab* genotype is a mechanism of *H*. *pylori* adaptation [33, 41]. To try and gain a better understanding of these adaptations, we asked if there were associations among the *bab* loci. In both populations, *babB* was more likely to be expressed from locus B when *babA* occupied locus A. Conversely, if *babB* was expressed from locus A then the *babB* at locus B was more likely to be ‘OUT’ of frame. Through these analyses we also noted that the *babC* gene was far more likely to occur in strains that also carry a *babB*. Furthermore, in strains containing both *babB* and *babC*, *babB* was more likely to be ‘OUT’ of frame. Taken together, these data suggest that *H*. *pylori* actively adjusts its repertoire of *bab* genes, not only through gene replacement, but also through the formation of chimeric proteins and phase variation supporting the finding of other authors [35, 42]. Although the function of *babB* and *babC* are unknown, it could be speculated that the Bab OMPs share overlapping functions, perhaps acting as adhesins similar to BabA. The differences between *babA*, *babB*, and *babC* may affect their function. For instance, if they are functioning as adhesins, these differences may result from different binding targets. Alternatively, the difference between these three genes may also make them antigenically distinct and allow them to escape the immune system.

To this end, we expanded our analysis to include other OMPs from the Hom family. There were no statistically significant differences when we compared strains carrying *homA* to those carrying *homB*; however, given that *homA* and *homB* are greater than 90% identical we decided to ask whether or not we saw an difference in *vacA*, *cagA* and *bab* genotypes between strains carrying a single *hom* gene at locus A or locus B versus strains in which both loci were occupied, regardless of whether it was *homA* or *homB*. We analyzed each population individually as well as combined, and observed an association between the *bab* gene carried at locus A and number of *hom*A and/or B genes. In both population, *babA* occurred most frequently with a single *homA* or *homB* gene, *babB* occurred relatively equally whether a single or both *hom* loci were occupied, and *babC* was only found in strains with a single *homA* or *homB* gene. If one considers the environment in which *H*. *pylori* persist, having a variable repertoire of OMPs is likely paramount to the success of the organism. While *H*. *pylori* has greater than 50 OMPs, to our knowledge this is the first evidence showing an association between the Hom and Bab families [16, 37, 45]. Since only the function of *babA* is known, it is difficult to speculate how these proteins may interact functionally [33]; however, one can surmise that *H*. *pylori* finds the right combination of OMPs as a means to balance between the adherent population, which is close to nutrients, and a free population, which is less susceptible to immune clearance. Through mechanisms such as phase variation and gene conversion *H*. *pylori* is able to fine tune its OMP repertoire in response to changes in the environment.

In addition to investigating the relationship between the *bab* genes and other OMPs, we were also interested in how *bab* genotypes related to known virulence factors such as *cagA* and *vacA* in KH [11, 12, 22, 46] and AH (S1A Table). In case of *cagA* genotypes, interestingly the AH group contained none of the EPIYA-D motif while KH included 7 strains possessing the EPIYA-C motifs. Importantly, we discovered several associations. A previous study found that strains with *babA* were more likely to have a *cagA* gene than strains that lacked *babA* [20]. While all of the strains in our collection contained a *cagA* gene [22], we did find that there was a positive association between having *babA* at locus A and having a *cagA* EPIYA-ABD motif. Strains that carried *babB* or *babC* at locus A were significantly more likely to have a non EPIYA-ABD motif. Carrying a *babA* at locus A was also significantly more likely to occur with *vacA* type s1/i1/m1. Our group has previously shown that *cagA* EPIYA-ABD and *vacA* s1/i1/m1 are associated with more severe disease in the KH [11]. Our current data suggest that carriage of *babA* at locus A may also be associated with these more virulent genotypes. Furthermore, we observed a three-way association between *bab* genotype at locus A, occupation of one or both *hom* loci and the *vacA* s1/i1/m1 genotype. Based on these three-way associations, we observed some interesting patterns. For instance, strains harboring a single *homA* or *homB* gene were more likely to be associated with *vacA* s1, i1 and m1 if there was a *babA* at locus A; however, if there was a *babB* at locus A, then strains with a single *homA* or *homB* gene were more likely to be found with a strain possessing s2/i2/m2, supporting the idea that *babA* may be associated with more virulent genotypes. However, if both *hom* loci are occupied and *babB* is at locus A, then this strain was more likely to be associated with s1, i1, and m1. These findings perhaps demonstrate that it is a specific combination of virulence factors that combine to result in more severe disease outcomes. Although we did not see higher order associations with disease, it could be that our sample size was not adequate to obtain statistical significance over the number of variables that were included.

Taken *en masse*, the *H*. *pylori* isolates obtained from the American population showed a larger degree of diversity in *bab* genotypes compared to the South Korean population. This may reflect adaptions to a more diverse human population. Furthermore, difference seen between the South Korean and American isolates due to *babB* phase variation may also be due to differences in the populations. Given that difference in host genetics are known to exist, perhaps *babB* is beneficial to *H*. *pylori* in the South Korean population but not in the American population. Even though the populations were distinct, we identified associations that were seen in both populations, as well as associations that became more apparent when we combined the populations and increased the sample size. Based on our analyses it appears that *babA* may be associated with more virulent genotypes of *cagA* and *vacA*. We also identified an association between OMPs from the Hom family of proteins with the *bab* genes, suggesting a potential coordination between OMP families. Interestingly, our phylogenetic analysis suggests that the using only the *hom* and *bab* genotypes segregated strains as well as the full genotyping scheme. Specifically the *hom* genotype appears to influence whether the strains cluster with AH or KH. Previous studies have demonstrated that strains with the same *cagA* EPIYA type cluster geographically [46]; however, to our knowledge this is the first report in which the number and type of OMP genes have been shown to segregate strains geographically. Although *homA* and *homB* sequence variation has been shown to cluster strains [51], the prior work focused on strains with a single *homA* or *homB* gene, whereas our study focused on which *hom* loci were occupied and not sequence differences. Thus, our observations suggest that perhaps in addition to geographic variation of *homA* and *homB* sequence, there is also a geographic difference in location of the *hom* genes, as well as number of loci occupied (1 vs. 2). Furthermore, these data suggest that OMPs may be a valuable tool to help develop a comprehensive classification system to identify *H*. *pylori* populations and sub-populations. In all, this study has expanded the profile of *bab* possible genotypes and characterized a new population. Furthermore, it highlights several associations that should be investigated next at a functional level.

## Supporting Information

S1 Fig Comparison of the presence of *bab* genes at loci A, B and C between 80 AH and 80 KH. (TIF)

S2 Fig Status of *babB* reading frame. (A) Percentage of ‘IN’ and ‘OUT’ *babB* gene reading frame in each population. (B) Number of ‘IN’ and ‘OUT’ *babB* gene reading frame at each locus.
(TIF)

S3 Fig Alignment of the amino acid sequences of 13 BabCs and 1 BabBC (AH-J68). Variable regions 1 and 2 are indicated, VR1 and VR2, respectively, above the BabC amino acid sequence.
(PDF)

S1 Table A. *cagA* EPIYA polymorphism, *vacA* s/i/m polymorphism and *homA/B* genotype of 80 AH. B. *cagA* EPIYA polymorphism, *vacA* s/i/m polymorphism and *homA/B* genotype of 80 KH. (XLSX)

S2 Table A. *bab* genotype of 80 AH. B. *bab* genotype of 80 KH. (XLSX)

S3 Table Discrete character code for phylogenetic analysis. (XLSX)
