# Correction: *Helicobacter pylori bab* Paralog Distribution and Association with *cagA*, *vacA*, and *homA/B* Genotypes in American and South Korean Clinical Isolates

**DOI:** 10.1371/journal.pone.0176468

**Published:** 2017-04-20

**Authors:** Aeryun Kim, Stephanie L. Servetas, Jieun Kang, Jinmoon Kim, Sungil Jang, Ho Jin Cha, Wan Jin Lee, June Kim, Judith Romero-Gallo, Richard M. Peek, D. Scott Merrell, Jeong-Heon Cha

Upon review of the published manuscript, four typographical errors in the underlying dataset were identified.

Two errors occurred in the labeling of the disease status of the AH clinical isolates in S1 Table; J262 was isolated from a patient with CA and J300 was isolated from a patient with BE. Due to the mislabeled disease states, a sentence within the “Sample Population” sub-header in the “Results” section should be amended to read as follows: Of these clinical isolates, 8.8% were from patients with cancer/pre-malignant lesions (of those, 2.5% were gastric carcinoma and 6.3% were Barrett's Esophagus), 43.7% were from patients with peptic ulcer disease (of those 31.2% were duodenal ulcers and 12.5% were gastric ulcers), 32.5% were from patients with gastritis, and 15.0% were from patients with esophagitis.

Next, the *cagA* EPIYA type of B130A should be reported as AB instead of ABCC in S1 Table, this lead to a miscoding for strain B130A listed in S3 Table.

Finally, the *bab* genotype for B130A is *babA/babB/babAB* but locus C was miscoded in S3 Table; therefore, S3 Table has been updated to reflect *babAB* at locus C.

The authors have re-run all statistical analyses. *P*-values that need to be adjusted are indicated in red in the corrected Tables 3 and 4. Despite slight variations to *P*-values listed in the corrected Tables 3 and 4, all significant associations remain significant and non-significant associations are also unchanged. In addition, the phylogenetic analysis was also re-run to accommodate updates to S3 Table. To account for updates to the analysis programs (figtree and Phylip), the authors ran the correct and uncorrect datasets side-by-side following the same parameters listed in the materials and methods. The results showed very similar distributions for both data sets and the significant findings did not change. Therefore, no changes to figure 4 are needed.

**Table 3:** Significant two-way comparisons of *bab* genotype and other factors in both populations^a^.

*bab* genotype comparison	*P* value^b^ for distribution within		
	KH	AH	Combined^c^
*babA*,*B*,C or empty at Locus A vs *babA*,*B*,*C* or empty at Locus B	0.1046	**0.0028**	**0.0131**
*babA*,*B*,C or empty at Locus A vs Full or empty *bab* at Locus C	**0.0027**	0.4111	0.4500
*babA*,*B*,C or empty at Locus A vs one or two *hom* loci occupied	**0.0167**	**<0.0001**	**0.0029**
*babB* ‘IN’ or Other at Locus B vs one or two *hom* loci occupied	**0.0210**	1.0000	0.6070
Full or empty *bab* at Locus C vs one or two *hom* loci occupied	1.0000	**0.0390**	0.1750
*babA*,*B*,C or empty at Locus A vs *homA or homB*	0.6390	**<0.0001**	**<0.0001**
*babA*,*B*,*C* or empty at Locus B vs *homA* or *homB*	0.7340	**0.0160**	**0.0260**
*babA*,*B*,C or empty at Locus A vs v*acA* s1 or *vacA* s2	1.0000	**<0.0001**	**<0.0001**
*babA*,*B*,C or empty at Locus A vs v*acA* i1 or *vacA* i2	0.9999	**<0.0001**	**<0.0001**
*babA*,*B*,C or empty at Locus A vs v*acA* m1 or *vacA* m2	0.9999	**<0.0001**	**<0.0001**
*babA*,*B*,C or empty at Locus A vs *cagA* EPIYA-ABD or Other	0.5010	N/A*	**<0.0001**
*babA*,*B*,*C* or empty at Locus B vs Cancer/Gastric ulcer or Duodenal ulcer/Gastritis	**0.0101**	0.974^#^	0.243^#^
*babB* or other at Locus B vs Cancer or Gastric ulcer or Duodenal ulcer or Gastritis	**0.0012**	0.225^#^	0.137^#^

^*a*^ For simplicity, Table 3 only contains associations for which a statistically significant association was found in at least one grouping; however, an exhaustive analysis was conducted on numerous other permutations of the data.

^*b*^ Statistically significant *P* values are in boldface type.

^*c*^ All isolates (n = 160) analyzed as a single group

*No ABD in the AH

^#^Also includes a category for Esophagitis/Barrett's Esophagus

Red cells indicate corrected *P*-values.

**Table 4:** Three-way comparisons of *bab* genotype and other factors in both populations.

*bab* genotype comparison	*P* value^a^ for distribution within		
	KH	AH	Combined^b^
*babA*, *B* or *C* at Locus A vs *vacA* i1/i2 vs one or two *hom* loci occupied	N/A	0.097	**0.001**
*babA*, *B* or *C* at Locus A vs *vacA* s1/s2 vs one or two *hom* loci occupied	N/A	0.173	**0.002**
*babA*, *B* or *C* at Locus A vs *vacA* m1/m2 vs one or two *hom* loci occupied	N/A	0.882	**0.001**
*babA*, *B* or *C* at Locus A vs *vacA* s1i1m1/other vs one or two *hom* loci occupied	1.000	N/A^#^	N/A^#^
*babA*, *B* or *C* at Locus A vs *cagA* (AB &Other^$^/ABCs/ABD) vs *vacA* m1/m2	N/A	0.972	0.075
*babA*, *B* or *C* at Locus A vs *cagA* (AB &Other^$^/ABCs/ABD) vs *vacA* s1/s2	N/A	0.781	0.666
*babA*, *B* or *C* at Locus A vs *cagA* (AB &Other^$^/ABCs/ABD) vs *vacA* i1/i2	N/A	0.571	0.075
*babA*, *B* or *C* at Locus A vs *cagA* (Other/ABD) vs *vacA* s1i1m1/other	1.000	N/A^#^	N/A^#^
*babA*, *B* or *C* at Locus A vs *cagA* (AB &Other^$^/ABCs/ABD) vs one or two *hom* loci occupied	0.976	0.997	**0.042**
*babA*, *B* or *C* at Locus B vs one or two *hom* loci occupied vs *vacA* s1/s2	N/A*	0.823	0.061

^*a*^ Statistically significant *P* values are in boldface type.

^*b*^ All isolates (n = 160) analyzed as a single group.

^#^These comparisons were only done in KH; for AH and combined we looked at i, s, and m regions of *vacA* separately, which wasn’t feasible with KH since was overwhelmingly s1/i1/m1.

*All KH strains are *vacA* s1.

^$^AB&Other refers to any *cagA* EPIYA motif that is not ABC_(1–4)_, or ABD.

Red cells indicate corrected *P*-values.

Please see the correct Tables 3 and 4 below. The corrected S1 and S3 Tables are attached as supporting information files.

## Supporting information

S1 Table A. *cagA* EPIYA polymorphism, *vacA* s/i/m polymorphism and *homA/B* genotype of 80 AH. B. *cagA* EPIYA polymorphism, *vacA* s/i/m polymorphism and *homA/B* genotype of 80 KH.
(XLSX)

S3 Table Discrete character code for phylogenetic analysis. (XLSX)
