# Supplementary figures and images for: Helicobacter pylori bab Paralog Distribution and Association with cagA, vacA, and homA/B Genotypes in American and South Korean Clinical Isolates
Source: PLoS One. 2015 Aug 28;10(8):e0137078. doi: 10.1371/journal.pone.0137078 (PMC4552749; doi:10.1371/journal.pone.0137078)

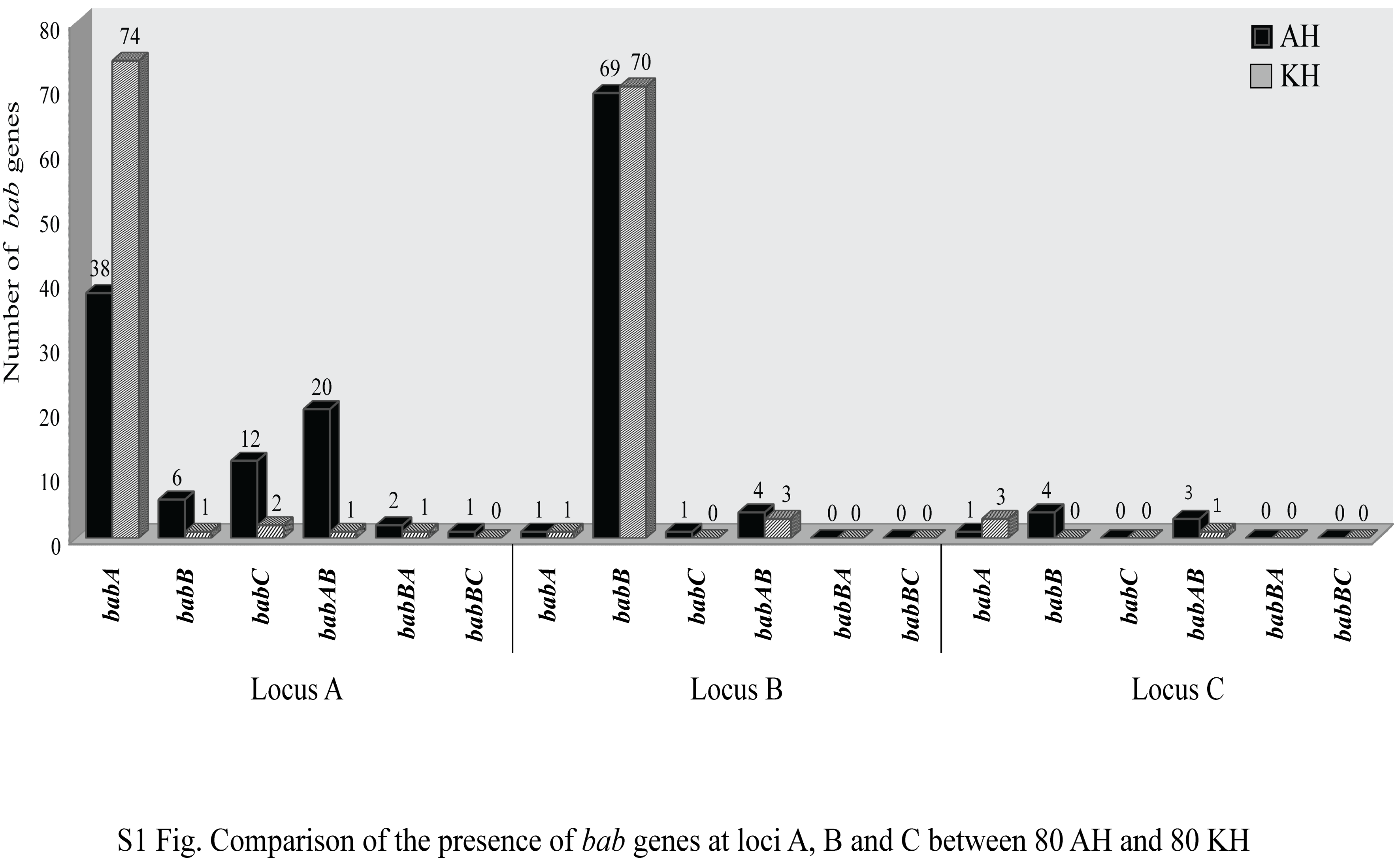

Supplement: S1 Fig — (TIF) [file pone.0137078.s001.tif]

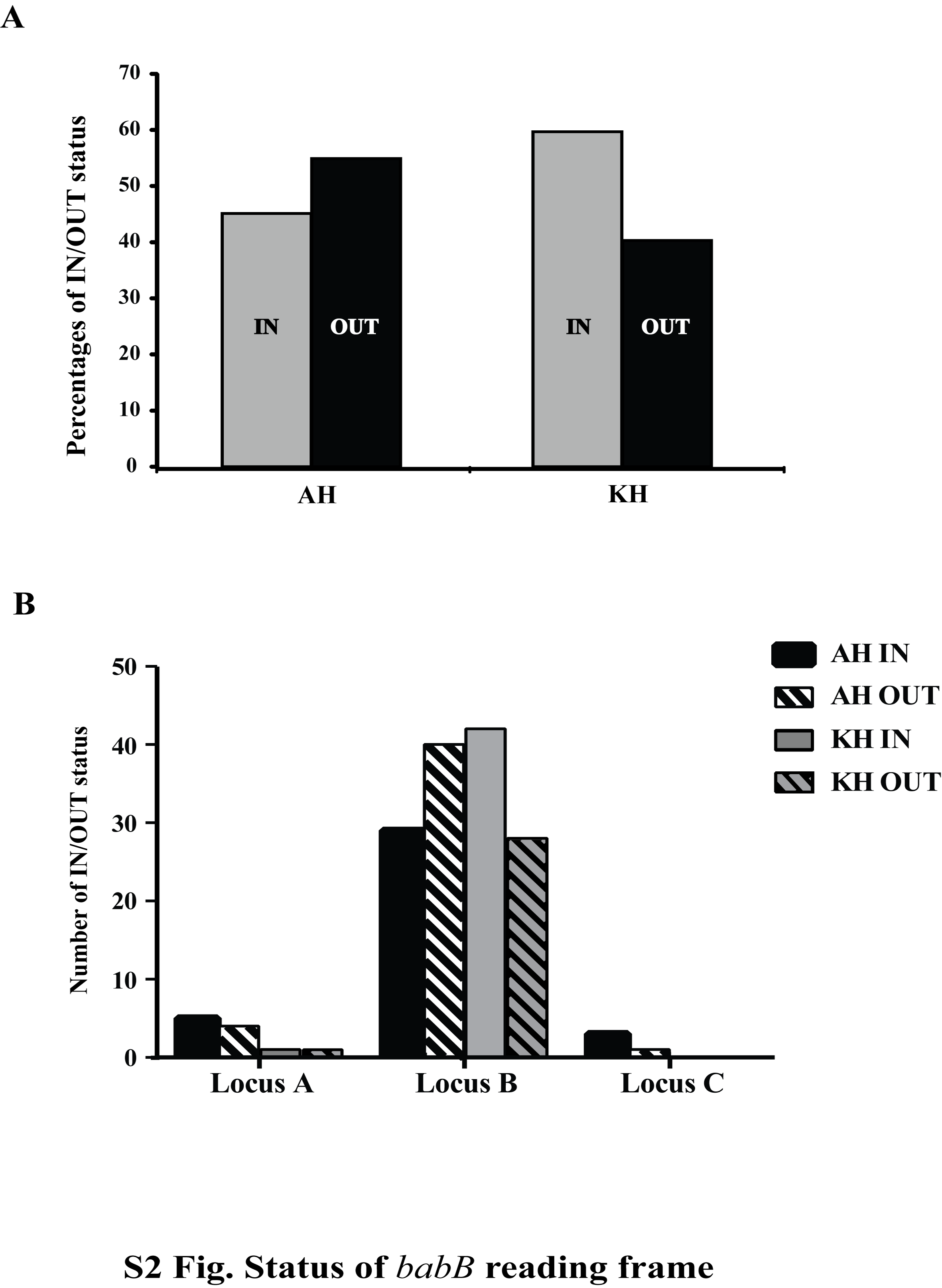

Supplement: S2 Fig — (A) Percentage of ‘IN’ and ‘OUT’ babB gene reading frame in each population. (B) Number of ‘IN’ and ‘OUT’ babB gene reading frame at each locus. (TIF) [file pone.0137078.s002.tif]
