# Supplementary material for: Helicobacter pylori bab Paralog Distribution and Association with cagA, vacA, and homA/B Genotypes in American and South Korean Clinical Isolates
Source: PLoS One. 2015 Aug 28;10(8):e0137078. doi: 10.1371/journal.pone.0137078 (PMC4552749; doi:10.1371/journal.pone.0137078)
Supplement: S3 Fig — Variable regions 1 and 2 are indicated, VR1 and VR2, respectively, above the BabC amino acid sequence. (PDF) [file pone.0137078.s003.pdf]

	1 … 50 … 100
**G27-babC**	MKKHILSLALGSLLVSALSAEDDGFYTSVGYQIGEAAQMVTNTKGIQELSDRYESLNNLLTRYSTLNTLIKLSADPSAVSGAINNLNAGATGLLKEKTNS
**KH-k17**	MKKHILSLALGSLLVSALSAEDDGFYTSVGYQIGEAAQMVTNTKGIQELSDRYESLNNLLTRYSTLNTLIKLSADPSAVSGAINNLNAGATGLLKEKTNS
**KH-k241**	MKKHILSLALGSLLVSALSAEDDGFYTSVGYQIGEAAQMVTNTKGIQELSDRYESLNNLLTRYSTLNTLIKLSADPSAVSGAINNLNAGATGLLKEKTNS
**AH-J58**	MKKHILSLALGSLLVSTLSAEDDGFYTSVGYQIGEAAQMVKNTKGIQQLSDNYEKLNNLLNNYSTLNTLIKLSADPSAVSGAINNLNAGATGLLKEKTNS
**AH-J68**	MKKT---LLLSLSLSFLLHAEDDGFYTSVGYQIGEAAQMVKNTKGIQQLSDNYEKLNNLLNNYSTLNTLIKLSADPSAISGAINNLNAGATGLLKEKTNS
**AH-J44**	MKKHILSLTLGSLLVSTLSAEDDGFYTSVGYQIGEAAQMVTNTKGIQELSDNYEKLNNLLTRYSTLNTLIKLSADPSAVSGAINNLNAGATGLLKEKTNS
**AH-J75**	MKKHILSLTLGSLLVSALSAEDDGFYTSVGYQIGEAAQMVTNTKGIQELSDNYEKLNNLLTRYSTLNTLIKLSADPSAVSGTINNLNAGATGLLKEKTNS
**AH-B147**	MKKHILSLTLGSLLVSTLSAEDDGFYTSVGYQIGEAAQMVKNTKGIQELSDNYEKLNNLLNSYSTLNTLIKLSADPSAVSGAINNLNAGATGLLKEKTNS
**AH-B175A**	MKKHILSLTLGSLLVSTLSAEDDGFYTSVGYQIGEAAQMVTNTKGIQELSDNYEKLNNLLNSYSTLNTLIKLSADPSAVSGAINNLNAGATGLLKEKINS
**AH-B201A**	MKKHILSLTLGSLLVSTLSAEDDGFYTSVGYQIGEAAQMVTNTKGIQELSDNYEKLNNLLNRYSTLNTLIKLSADPSAVSGAINNLNAGATGLLKEKTNS
**AH-B253A**	MKKHILSLTLGSLLVSALSAEDDGFYTSVGYQIGEAAQMVKNTKGIQELSENYEKLNNLLTKYSTLNTLIKLSADPSAVSGAINNLNAGATGLLKEKTNS
**AH-B269A**	MKKHILSLTLGSLLVSALSAEDDGFYTSVGYQIGEAAQMVTNTKGIQELSDNYEKLNNLLTRYSTLNTLIKLSADPSAVSGAINNLNAGATGLLKEKTNS
**AH-B279A**	MKKHILSLALGSLLVSTLSAEDDGFYTSVGYQIGEAAQMVTNTKGIQDLSDRYESLNNLLTKYSTLNTLIKLSADPSAVSGAINNLNAGATGLLKEKENS
**AH-B212A**	MKKHILSLTLGSLLVSALSAEDDGFYTSVGYQIGEAAQMVTNTKGIQELSDNYEKLNNLLTRYSTLNTLIKLSADPSAVSGAVNNLNAGATGLLEEKTNS
**AH-B234A**	MKKHILSLTLGSLLVSTLSAEDDGFYTSVGYQIGEAAQMVTNTKGIQELSDNYEKLNNLLTKYSTLNTLIKLSADPSAVSGAINNLNAGATGLLKEKTNS
**AH-B301A**	MKKHILSLALGSLLVSTLSAEDDGFYTSVGYQIGEAAQMVTNTKGIQQLSDNYEKLNNLLTRYSTLNTLIKLSADPSAVSGAINNLNAGATGLLKEKINS
**Consensus**	MKKHILSLTLGSLLVSTLSAEDDGFYTSVGYQIGEAAQMVTNTKGIQELSDNYEKLNNLLTRYSTLNTLIKLSADPSAVSGAINNLNAGATGLLKEKTNS

	101 … 150 … 200
**G27-babC**	PAYQAVSLALNAAVGLWNTIGYAVMCGNGNGTEGGPGSVVFSNEPGQKSTQITCNRYEATGPGKSMSIDEFKKLNEAYQIIQQALKNQHGFPELGGNSTK
**KH-k17**	PAYQAVSLALNAAVGLWNTIGYAVMCGNGNGTEGGPGSVVFSNEPGQKSTQITCNRYEATGPGKSMSIDEFKKLNEAYQIIQQALKNQHGFPELGGNSTK
**KH-k241**	PAYQAVSLALNAAVGLWNTIGYAVMCGNGNGTEGGPGSVVFSNEPGQKSTQITCNRYEATGPGKSMSIDEFKKLNEAYQIIQQALKNQHGFPELGGNSTK
**AH-J58**	PAYQAVSLALNAAVGLWNTIGYAVMCGNGNDTGSGPGSVIFNEQPGQGSTTIICNRYESVGPGKSMSIEEFKKLNEAYQIIQQALKQQNGFPELGQNGTS
**AH-J68**	PAYQAVSLALNAAVGLWNTIGYAVMCGNGNGTGGGPGSVIFNGQPGQNSTAITCNRFEATGPGKSMSIDEFKKLNEAYQIIQQALKEGNGFPVLDGEGTK
**AH-J44**	PAYQAVSLALNAAVGLWNTIGYAVMCGNGNGTGGGPGSVVFNDQPGQDSTQITCNRYESTGPGKSMSIPEFKKLNEAYQIIQQALKKQTGFPELGGNGTS
**AH-J75**	PAYQAVSLALNAAVGLWNTIGYAVMCGNGNGTGGGPGSVIFDGQPGQGSTSITCNRFEATGPGKSMSIDEFKKLNEAYQIIQQALKKQRGFPELGESGTS
**AH-B147**	PAYQAVSLALNAAVGLWNTIGYAVMCGNGNGTGSGPGSVVFNEEPGQKSTAITCNRFESTGPGKSMSIDEFKKLNEAYQIIQQALKNQSGFPELGSNGTE
**AH-B175A**	PAYQAVFLALNAAVGLWNTIGYAVMCGNGNGTGSGPGSVIFNNQPGQSSTNITCNRFEPTGPGKSMSIDEFKKLNKAYQIIQQALKEGKGFPELGENGTT
**AH-B201A**	PAYQAVSLVLNAAVGLWNTIGYAVMCGNGNGTDSGPGSVIFNKQPGQGSTKITCNRYEATGPGKSMSIDEFKKLNEAYQIIQQALKDQNGFPELGGNGTS
**AH-B253A**	PAYQAVSLALNAAVGLWNTIGFAVMCGNGNDTDSGPGSVVFNGEPGEHSTAITCNRFESTGPGRSMSIPEFKKLNEAYQIIQQALNRQNGFPELGNNGTK
**AH-B269A**	PAYQAVSLALNAAVGLWNTIGYAVMCGNGNGTGSGPGSVIFNGEPGQGSTAITCNRFEATGPGKSMSIDEFKKLNEAYQIIQQALKKGSGFPELGGNGTN
**AH-B279A**	PAYQAVSLALNAAVGLWNTIGYAVMCGNGNGTGSGPGSVIFNGQPGQGSTQITCNRFESTGPGKSMSIDEFKKLNEAYQIIQQALKTQSGFPELGENGKN
**AH-B212A**	PAYQAVFLALNAAVGLWNTIGYAVMCGNGNGTGGGPGRVIFNGQPGAHSTHIICDRYEATGPGKSMSIDEFKKLNEAYQIIQQALKNRDGFPELGGKGTS
**AH-B234A**	PAYQAVSLALNAAVGLWNTIGYAVMCGNGNNTGGGPGSVIFKGQPGQNSTAITCDRYESTGPGKSMTIDEFKKLNEAYQIIQQALKGG-GFPELGNSGTK
**AH-B301A**	PAYQAVSLALNAAVGLWNTIGYAVMCGNGNDTGSGPGSVVFSGEPGENSTQITCNRYESTGPGKSMTIDEFKKLNEAYQIIQQALERGNGFPELGGNGTK
**Consensus**	PAYQAVSLALNAAVGLWNTIGYAVMCGNGNGTGSGPGSVIFNGQPGQ ST ITCNRYEATGPGKSMSIDEFKKLNEAYQIIQQALK Q GFPELGGNGT

```
                201                    -VR1-                   250                                      300
G27-babC        VTVEYEYECKQNNN--GNNINDGVNQFCRAKNGSSSSGS---NDTSTQTTTQDGVTITTTYSN----NKATVNFSITNNAQELLNQAANIMQVLNTQCPL
KH-k17          VTVEYEYECKQNNN--GNNINDGVNQFCRAKNGSSSSGS---NDTSTQTTTQDGVTITTTYSN----NKATVNFSITNNAQELLNQAANIMQVLNTQCPL
KH-k241         VTVEYEYECKQNNN--GNNINDGVNQFCRAKNGSSSSGS---NDTSTQTTTQDGVTITTTYSN----NKATVNFSITNNAQELLNQAANIMQVLNTQCPL
AH-J58          VSVNYHYECKQIN----SEINGGVNQFCQAKNGSSSNGSSSSNGTSTQTTTQDGVTITTKYKD----NKATVLFSITNNAQELLNQAANIMQVLNTQCPL
AH-J68          VTVKYTYECQEAKD---SKINNGVDQFCQAKNSSGSSNHNGSNGTSTQTTTQDGVAITTTYNN----NKATVNFSIINNAQQLLNQAANIMQVLNTQCPL
AH-J44          VNVEYGYECKQKDD-SDSSISGGVNQFCQAEHGS---GT---NGNSTTTTTQDGVAITTTYHN----NNATVNFQIINNAQELLNQAANIMQVLNTQCPL
AH-J75          VNVSYKYECKQENR-----TNGGVSQFCQAKSQAKNGSS---NGNGTTTTIQDGVTITTTYNN---KDKATVSFGITNNAQQLLNQAANIMQVLNTQCPL
AH-B147         VKVNYTYECKQTS-----NINGGVNQFCQK-NGSS-----GSNSTSTQTTNQDGVTITTTYND----HKATVNFNITNNAQELLNQAANIMQVLNTQCPL
AH-B175A        VNVKYEYECEQQTD---S-INGGVNQFCQAKNGSGS------NSTSKQMTNQDGVAITTIYNN----NKAIVTFSITNNAQQLLNQAANIMQVLNTQCPL
AH-B201A        VNITYEYKCEQT-----ADINGGVSQFCRAKNGSS--GSNGSNGTSTQITTQDGVTITTTYSD----NNATVNFSITNNAQELLNQAANIMQVLNTQCPL
AH-B253A        VNVKYEYECKQKNQ----AINGGVNQFCEAKNGSS-----G---TSTQTTTQDGVTITTTYGG----NKATVEFDITNNAEQLLNQAANIMQVLNTQCPL
AH-B269A        VNVNYTYECRHNNNNNDNNINGGVNQFCQAKNGSSGTSGSDSNSSSTQTTTQDGVMITTTYSD----NKATVNFSITNNAEQLLNQAANIMQVLNTQCPL
AH-B279A        VNVSYEYECKQTS-----DINGGVDQFCQVKNGSSGTG--------TQTTNQDGVAITTTYNN----NKATVNFNITNNAQQLLNQAANIMQVLNTQCPL
AH-B212A        VSVSYEYECKQSNDSNDSSINGGVNQFCQTKNGSS-----GSNGSDVQTTTQDGVKIITEYRN----NKATVNFSITNNAQQLLNQAANIMQVLNTQCPL
AH-B234A        VNVEYEYECNQKNN-----INGGVDQFCRAKNGSSSSSG-TNGTSTQTTTQDGVTITTTYHNNNNNNSATVKFDITNNAEQLLNQAANIMQVLNTQCPL
AH-B301A        VEIKYNYECNQTANNQDANINGGVNQFCKAKN-----------GNTTQTTTQDGVTITTTYNN----NKAIVNFSITNNAQQLLAQAANIMQVLNTQCPL
Consensus       VNV YEYECKQ      N INGGVNQFCQAKNGSS S     NGTSTQTTTQDGVTITTTY N    NKATVNFSITNNAQQLLNQAANIMQVLNTQCPL
```

```
                301                                              350                                                400
G27-babC        VRSTHDENNAGNGNPWGLSTSGNACQIFQQEFSQVTSMIKNAQEIVAQSQIANANQKAEIANPSNFNPFTDASFAQSMLKNARAQAEMFNLAEQVKKNLE
KH-k17          VRSTHDENNAGNGNPWGLSTSGNACQIFQQEFSQVTSMIKNAQEIVAQSQIANANQKAEIANPSNFNPFTDASFAQSMLKNARAQAEMFNLAEQVKKNLE
KH-k241         VRSTHDENNAGNGNPWGLSTSGNACQIFQQEFSQVTSMIKNAQEIVAQSQIANANQKAEIANPSNFNPFTDASFAQSMLKNARAQAEMFNLAEQVKKNLE
AH-J58          VRSMHDVNAKGDGQPWSLSTTGNACTIFQQEFSQVTSMIKNAQEIVAQSKIANANQKAEISNPSNFNPYTDASFSQSMLKNARAQAEMFNLAEQVKKNLE
AH-J68          VRSTSNENRPGNGSPWGLTTGGDACQIFQQEFSQVTSMIKNAQEIIAQSKIANAKQQAEIANPSNFNPFTDASFAQSMLKNARAQAEMFNLSEQVKKNLE
AH-J44          VRSSHDQNASGDGRPWGLSTSGNACQIFQQEFSQVTSMIKNAQEIVAQSQIANANQKAEISNPSNFNPFTDASFAQGMLKNAQAQAEMFNLSEQVKKNLE
AH-J75          VRSKNNENVQGDGSPWGLSTSGDACQIFQQEFSQVTNMIKNAQEIVAQSQIANANQNAEIANPSNFNPFTDASFAQSMLKNARAQASMFNLAEQVKKNLE
AH-B147         VRSMYNENVPGGGQPWSLSTSGDACQIFQQEFSQVTSMIKNAQEIVAQSKIANANQNAEIHNPSNFNPFTDASFAQDMLKNARAQAEMFNLAEQVKKNLE
AH-B175A        VRSRHDENNAGDGSPWGLSTSGNACTIFKQEFSQVTSMIKNAQEIVVQSEIANTNQNAQIHNPSNFNPFTDASFAQGMLANARAQAEMFNLAEQVKKNLE
AH-B201A        VRSRHNENASGDGSPWGLSTSGDACQIFQQEFSQVTSMIKNAQEIVAQSKIANANQQAEIANPSNFNPFTDASFAQDMLKNARAQAEMFNLAEQVKKNLE
AH-B253A        VRSTNNENKPGGGQPWGLSTHGNACQIFQQEFSAVTSMIKNAKEIVAQSKIANANQKVEIANPSNFNPFTDASFAQDMLKNARAQAEMFNLAEQVKKNLE
AH-B269A        VRSTHDVNASGDGQPWGLTTSGDACQIFQQEFSQVTSMIKNAQEIVAQSKIANANQQAEIHNPSNFNPFTDASFAQSMLKNARAQAEMLNLAEQVKKNLE
AH-B279A        VRSTNDVNTPGGGQPWGLSTSGDACQIFQQEFSQVTSMIKNAQEIVAQSKIANANQQAEISNPSNFNPFTDASFAQDMLKNARAQASMFNLAEQVKQNLE
AH-B212A        VRSMYHVNNVGDGSPWGLSTSGNACTIFQQEFSQVTSMIKNAQEIVAQSQIANANRRAEISNPSNFNPFTDASFAQSMLKNARAQAEMFNLAEQVKKNLE
AH-B234A        VRSTHSENAQGDGKPWGLSTSGNACQIFQQEFSQVTSMIKNAQEIVAQSKIANNNQQAEIANPSSFNPYTDAGFAQSMLKNAQAQASMFNLAEQVKKNLE
AH-B301A        VRSTNNANAPGDGRPWGLSTSGNACQIFQQEFSQVTSMIKNAQEIVAQSKIANANQKAEITNPSNFNPFTDASFAQSMLKNAQAQAEMFNLAEQVKKNLE
Consensus       VRSTHDEN  GDGQPWGLSTSGNACQIFQQEFSQVTSMIKNAQEIVAQSKIANANQQAEIANPSNFNPFTDASFAQSMLKNARAQAEMFNLAEQVKKNLE
```

```
          401    |  -VR2-  |                        450                                          500
G27-babC  VMQNNNNVNAELSGFGKGMTNFVSAFLASCRNGGGTLPNEGVTSNTWGAGCAYVGETISALNNSIAHFGTQEQQIQQAENIADTLVNFKSRYSELGNTYN
KH-k17    VMQNNNNVNAELSGFGKGMTNFVSAFLASCRNGGGTLPNEGVTSNTWGAGCAYVGETISALNNSIAHFGTQEQQIQQAENIADTLVNFKSRYSELGNTYN
KH-k241   VMQNNNNVNAELSGFGKGMTNFVSAFLASCRNGGGTLPNEGVTSNTWGAGCAYVGETISALNNSIAHFGTQEQQIQQAENIADTLVNFKSRYSG------
AH-J58    VMQNNNNV-KE----GKGTTNFVSAFLANCHNGGGTLPNAGVTSNTWGAGCAYVGETITALNNSIAHFGTEAQQIQQAADIADTLVNFKSRYSELGNTYN
AH-J68    VMQNNNNVNKKLSGFGEGMTNFVSAFLASCKGDG-TLPNEGVTFNTWGAGCAYVGETITALTNSIAHFGTQAEQIQQAENIADTLVNFKSRYSELGNTYN
AH-J44    VMQNNNNV----GGFGEKMTNFVSAFLASCKDGGGTLPNQGVTSNTWGAGCAYVQETITALNNSIAHFGTQAQQIQQAENIADTLVHFKSRYNELGNTYN
AH-J75    VMQNNNNVDANLSGFGKGMTNFVSAFLASCKDGG-TLPNEGVTSNTWGAGCAYVEETITALTNSIAHFGTQAEQIQQAENIADTLVNFKSRYSELGNTYN
AH-B147   VMQNNNNVII-----DGKATNFVSAFLATCKVDG-TLPTAGVTSNTWGAGCAYVGETITALNNSIAHFGTQEQQIEQAENIADTLVNFKSRYSELGNTYN
AH-B175A  VMQNNNNVNVNLSRLGERMTNFVSAFLASCRDDG-TLPDQGVTSNTWGAGCAYVGETITALNNSIAHFGTQAQQIQQAENIADTLVNFKSRYSELGNTYN
AH-B201A  VMENIT-V--TGY--GN-NQNFVSAFLASCKADG-TLPNHGVTSNTWGAGCAYVEETISTLNNSIAHFGTQEQQIQQAENIADTLVNFKSRYSELGNTYN
AH-B253A  VMKNNNNVTI-----EGKATNFVSAFLASCKADG-TLPNAGVTSNTWGAGCAYVGETITALTNSIAHFGTQEQQIQQAENIADTLVNFKSRYSELGNTYN
AH-B269A  VMQNNNNVNAELSGFGTGMTNFVSAFLASCKNGG-TLPNEGVTRSTWGAGCAYVGETITALNNSIAHFGTQAQQIQQAENIADTLVNFKSRYNELGNTYN
AH-B279A  VMENNNNVT----GFGSGMTNFVSAFLATCKVDG-TLPNAGVTSNTWGAGCAYVGETITALNNSIAHFGTQAQQIQQAENIADTLVNFKSRYNELGNTYN
AH-B212A  VMQNNNNVKI-----DGGLTNFVSAFLASCRHDG-TLPNAGVTNNTWGAGCAYVGETISALTNSIAHFGTQAEQIQQAENIADTLVNFKSRYSELGNTYN
AH-B234A  VMQNNNNVDAGLSGFGKGMTNFVSAFLASCKDGG-TLPDAGVTSDTWGAGCAYVQETITALTNSIAHFGTQAEQIQQAENIADTLVNFKSKYSELGNTYN
AH-B301A  VMQNNNNVNKKLSGFGEGMTNFVSAFLASCRDDG-TLPNAGVTSNTWGAGCAYVGETITALTNSIAHFGTQEQQIQQAKNIADTLVNFKSRYNELGNTYN
Consensus VMQNNNNV    LSGFG GMTNFVSAFLASCK GG TLPN GVTSNTWGAGCAYVGETITALNNSIAHFGTQAQQIQQAENIADTLVNFKSRYSELGNTYN

          501                                              550                                             600
G27-babC  SITTALSSIPNGSSLQNVVSKKNNPYSPQGIETNYYLNQNSYNQVQTINQELGRNPFRKVGIVSSQTNNGAMNGIGIQVGYKQFFGQKRKWGARYYGFFD
KH-k17    SITTALSSIPNGSSLQNVVSKKNNPYSPQGIETNYYLNQNSYNQVQTINQELGRNPFRKVGIVSSQTNNGAMNGIGIQVGYKQFFGQKRKWGARYYGFFD
KH-k241   ----------------------------------------------------------KVGIVSSQTNNGAMNGIGIQVGYKQFFGQKRKWGARYYGFFD
AH-J58    SITTALSSIPNAQSLQNAVSKKNNPYSPQGIETNYYLNQNTYNQIQTINQELGRNPFRKVGIVSSQTNNGAMNGIGIQVGYKQFFGQKRKWGARYYGFFD
AH-J68    SITTALSSIPNVQSLQNVVGKKNNPYSPQGIDTNYYLNQNAYNQIQTINQELGRNPFRKVGIVSSQTNNGAMNGIGIQVGYKQFFGQKRKWGARYYGFFD
AH-J44    SITTALSKVPNAQNLQNVVGKKNNPYSPQGIDTNYYLNQNSYNQIQTINQELGRNPFRKVGIVSSQTNNGAMNGIGIQVGYKQFFGQKRKWGARYYGFFD
AH-J75    SITTALSKVPNAQSLQNVVSKKNNPYSPQGIETNYYLNQNSYNQIQTINQELGRNPFRKVGIVSSQTNNGAMNGIGIQVGYKQFFGQKRKWGARYYGFFD
AH-B147   SITTALSKVPNAQSLQNAVSKKNNPYSPQGIETNYYLNQNSYNQIQTINQELGRNPFRKVGIVSSQTNNGAMNGIGIQVGYKQFFGQKRKWGARYYGFFD
AH-B175A  SITTALSSIPNAQSLQNAVNKKNNPYSPQGIETNYYLNQNSYNQIQTINQELGRNPFRKVGIVSSQTNNGAMNGIGIQVGYKQFFGQKRKWGARYYGFFD
AH-B201A  SITTALSSIPNAQSLQNAVSKKNNPYSPQGIETNYYLNQNSYNQIQTINQELGRNPFRKVGIVSSQTNNGAMNGIGIQVGYKQFFGQKRKWGARYYGFFD
AH-B253A  SITTALSKVPNAQSLQNAVSKKNNPYSPQGIDTNYYLNQNSYNQIQTINQELGRNPFRKVGIVSSQTNNGAMNGIGIQVGYKQFFGQKRKWGARYYGFFD
AH-B269A  SITTALSSIPNAQSLQNAVSKKNNPYSPQGIETNYYLNQNSYNQIQTINQELGRNPFRKVGIVSSQTNNGAMNGIGIQVGYKQFFGQKRKWGARYYGFFD
AH-B279A  SITTALSSIPNAQALQNAVSKKNNPYSPQGIETNYYLNQNTYNQIQTINQELGRNPFRKVGIVSSQTNNGAMNGIGIQVGYKQFFGQKRKWGARYYGFFD
AH-B212A  SITTALSSIPNAQSLQNAVSKKNNPYSPQGIETNYYLNQNSYNQIQTINQELGRNPFRKVGIVSSQTNNGAMNGIGIQVGYKQFFGQKRKWGARYYGFFD
AH-B234A  SITTALSNIPNAQSLQNVVSKKNNPYSPQGIETNYYLNQNSYNQVQTINQELGRNPFRKVGIVSSQTNNGAMNGIGIQVGYKQFFGQKRKWGARYYGFFD
AH-B301A  SITTALSKVPNAQSLQNVVGKKNNPYSPQGIDTNYYLNQNSYNQIQTINQELGRNPFRKVGIVSSQTNNGAMNGIGIQVGYKQFFGQKRKWGARYYGFFD
Consensus SITTALSSIPNAQSLQNAVSKKNNPYSPQGIETNYYLNQNSYNQIQTINQELGRNPFRKVGIVSSQTNNGAMNGIGIQVGYKQFFGQKRKWGARYYGFFD
```

```
                601                                              650                                                700
G27-babC        YNHAFIKSSFFNSASDVWTYGFGADALYNFINDKATNFLGKNNKLSVGLFGGIALAGTSWLNSEYVNLATMNNVYNAKMNVANFQFLFNMGVRMNLARSK
KH-k17          YNHAFIKSSFFNSASDVWTYGFGADALYNFINDKATNFLGKNNKLSVGLFGGIALAGTSWLNSEYVNLATMNNVYNAKMNVANFQFLFNMGVRMNLARSK
KH-k241         YNHAFIKSSFFNSASDVWTYGFGADALYNFINDKATNFLGKNNKLSVGLFGGIALAGTSWLNSEYVNLATMNNVYNAKMNVANFQFLFNMGVRMNLARSK
AH-J58          YNHAFIKSSFFNSASDVWTYGFGADALYNFINDKATNFLGKNNKLSVGLFGGIALAGTSWLNSEYVNLATVNNVYNAKLNVANFQFLFNMGVRMNLARSK
AH-J68          YNHAFIKSSFFNSASDVWTYGFGADALYNFINDKATNFLGKNNKLSVGLFGGIALAGTSWLNSEYVNLATVNNVYNAKMNVANFQFLFNMGVRMNLARPK
AH-J44          YNHAFIKSSFFNSASDVWTYGFGADALYNFINDKATNFLGKNNKLSVGLFGGIALAGTSWLNSEYVNLATMNNVYNAKMNVANFQFLFNMGVRMNLARPK
AH-J75          YNHAFIKSSFFNSASDVWTYGFGADALYNFINDKATNFLGKNNKLSVGLFGGIALAGTSWLNSEYVNLATMNNVYNAKMNVANFQFLFNMGVRMNLARPK
AH-B147         YNHAFIKSSFFNSASDVWTYGFGADALYNFINDKATNFLGKNNKLSVGLFGGIALAGTSWLNSEYVNLATVNNVYNAKMNVANFQFLFNMGVRMNLARPK
AH-B175A        YNHAFIKSSFFNSASDVWTYGFGADALYNFINDKATNFLGKNNKLSVGLFGGIALAGTSWLNSEYVNLATVNNVYNAKMNVANFQFLFNMGVRMNLARPK
AH-B201A        YNHAFIKSSFFNSASDVWTYGFGADALYNFINDKATNFLGKNNKLSVGLFGGIALSRHFMA*--------------------------------------
AH-B253A        YNHAFIKSSFFNSASDVWTYGFGADALYNFINDKATNFLGKNNKLSVGLFGGIALAGTSWLNSEYVNLATMNNVYNAKMNVANFQFLFNMGVRMNLARPK
AH-B269A        YNHAFIKSSFFNSASDVWTYGFGADALYNFINDKATNFLGKNNKLSVGLFGGIALAGTSWLNSEYVNLATMNNVYNAKMNVANFQFLFNMGVRMNLARPK
AH-B279A        YNHAFIKSSFFNSASDVWTYGFGADALYNFINDKATNFLGKNNKLSVGLFGGIALAGTSWLNSEYVNLATMNNVYNAKMNVANFQFLFNMGVRMNLARSK
AH-B212A        YNHAFIKSSFFNSASDVWTYGFGADALYNFINDKATNFLGKNNKLSVGLFGGIALAGTSWLNSEYVNLATVNNVYNAKMNVANFQFLFNMGVRMNLARPK
AH-B234A        YNHAFIKSSFFNSASDVWTYGFGADALYNFINDKATNFLGKNNKLSVGLFGGIALAGTSWLNSEYVNLATMNNVYNAKMNVANFQFLFNMGVRMNLARSK
AH-B301A        YNHAFIKSSFFNSASDVWTYGFGADALYNFINDKATNFLGKNNKLSVGLFGGIALAGTSWLNSEYVNLATMNNVYNAKLNVANFQFLFNMGVRMNLARPK
Consensus       YNHAFIKSSFFNSASDVWTYGFGADALYNFINDKATNFLGKNNKLSVGLFGGIALAGTSWLNSEYVNLATMNNVYNAKMNVANFQFLFNMGVRMNLARPK

                701                                              750
G27-babC        KKGSDHAAQHGIELGLKIPTINTNYYSFMGAELKYRRLYSVYLNYVFAY*
KH-k17          KKGSDHAAQHGIELGLKIPTINTNYYSFMGAELKYRRLYSVYLNYVFAY*
KH-k241         KKGSDHAAQHGIELGLKIPTINTNYYSFMGAELKYRRLYSVYLNYVFAY*
AH-J58          KKGSDHAAQHGIELGLKIPTINTNYYSFMGAELKYRRLYSVYLNYVFAY*
AH-J68          KKDSDHAAQHGIELGLKIPTINTNYYSFMGAELKYRRLYSVYLNYVFAY*
AH-J44          KKDSDHAAQHGIELGLKIPTINTNYYSFMGAELKYRRLYSVYLNYVFAY*
AH-J75          KKGSDHAAQHGIELGLKIPTINTNYYSFMGAELKYRRLYSVYLNYVFAY*
AH-B147         KKDSDHAAQHGIELGLKIPTINTNYYSFMGAELKYRRLYSVYLNYVFAY*
AH-B175A        KKDSDHAAQHGIELGLKIPTINTNYYSFMGAELKYRRLYSVYLNYVFAY*
AH-B201A        ---------------------------------------------------
AH-B253A        KKDSDHAAQHGIELGLKIPTINTNYYSFMGAELKYRRLYSVYLNYVFAY*
AH-B269A        KKDSDHVAQHGIELGLKIPTINTNYYSFMGAELKYRRLYSVYLNYVFAY*
AH-B279A        KKDSDHAAQHGIELGLKIPTINTNYYSFMGAELKYRRLYSVYLNYVFAY*
AH-B212A        KKDSDHAAQHGIELGLKIPTINTNYYSFMGAELKYRRLYSVYLNYVFAY*
AH-B234A        KKDSDHAAQHGIELGLKIPTINTNYYSFMGAELKYRRLYSVYLNYVFAY*
AH-B301A        KKDSDHAAQHGIELGLKIPTINTNYYSFMGAELKYRRLYSVYLNYVFAY*
Consensus       KKDSDHAAQHGIELGLKIPTINTNYYSFMGAELKYRRLYSVYLNYVFAY
```

S3 Fig. Alignment of the amino acid sequences of 13 BabCs and 1 BabBC (AH-J68).
